# Recent Advances in Conjugated Microporous Polymers for Photocatalysis: Mechanism, Synthesis and Applications

**DOI:** 10.1002/EXP.20240487

**Published:** 2025-12-22

**Authors:** Dun Zhou, Kongqing Zhang, Xiaobai Li, Yongpeng Liu, Liang Yao, Hongwei Ma

**Affiliations:** ^1^ College of Chemistry Chemical Engineering and Resource Utilization Northeast Forestry University Harbin P. R. China; ^2^ Heilongjiang Key Laboratory of Complex Traits and Protein Machines in Organisms Northeast Forestry University Harbin P. R. China; ^3^ Yusuf Hamied Department of Chemistry University of Cambridge Cambridge UK; ^4^ State Key Laboratory of Luminescent Materials and Devices Institute of Polymer Optoelectronic Materials and Devices Guangdong Basic Research Center of Excellence for Energy and Information Polymer Materials South China University of Technology Guangzhou P. R. China

**Keywords:** conjugated microporous polymers, photocatalysts, CO_2_ reduction, nanomaterials, solar energy conversion

## Abstract

As the global energy crisis intensifies and environmental pollution continues to deteriorate, harnessing solar energy through photocatalytic processes is emerging as a leading strategy to meet energy needs and combat climate crisis. Natural photosynthesis, the oldest and most fundamental photocatalytic system, converts sunlight, water, and CO_2_ into useful chemicals that sustain all forms of life. Inspired by this process, a range of artificial photosynthesis systems have been developed to store solar energy in chemical bonds. Conjugated microporous polymers (CMPs) have gained increasing attention as innovative photocatalysts, owing to their unique properties such as tunable light‐harvesting abilities, robust porous architectures, and extended π‐conjugated frameworks. This review outlines recent progress in the application of CMPs as photocatalysts, offering a comprehensive and critical analysis. It begins by introducing the evolution, preparation methods, and catalytic mechanisms of CMPs. The application of CMP‐based photocatalysis in hydrogen evolution, CO_2_ conversion, biomass valorization, and degradation of harmful substances are then described. Finally, future perspectives of CMPs as photocatalysts are presented, highlighting the key challenges and opportunities in their adaptation for solar energy conversion.

## Graphical abstract

Conjugated microporous polymers (CMPs) have attracted more and more attention as new photocatalysts due to their unique advantages such as high performance, long stability and strong light absorption. This review summarizes the application of CMPs as a photocatalyst in recent years and introduces the catalytic mechanism.

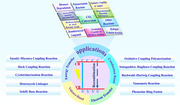

## 1 Introduction

As human civilization progresses, the growing dependence on fossil fuels has driven industrial growth while simultaneously leading to two pressing and interconnected issues—rising energy shortages and worsening climate change—both of which demand urgent and innovative solutions on a global scale [1, 2, 3]. In response, there has been a growing commitment to developing renewable energy sources, which offer promising strategies to address these pressing issues [4, 5]. Solar energy is considered the cleanest, most plentiful, and economically viable renewable resource, with the capacity to fulfill worldwide energy needs—provided it can be harnessed and stored efficiently. Natural photosynthesis, refined over millions of years of evolution, serves as an exceptional model for solar energy conversion and storage [6], which employs enzymes for selective catalysis and utilizes complementary light absorption in a Z‐scheme architecture [7, 8]. Inspired by this process, researchers have created artificial photosynthetic systems using synthetic materials to mimic and drive these processes [9, 10]. Fujishima and Honda pioneered photoelectrochemical water splitting in their seminal 1972 work, which used a TiO_2_ electrode to convert sunlight into chemical energy stored in H_2_ and O_2_ [11].

Although inorganic materials have advanced considerably, they still suffer from key limitations, including restricted light absorption, fast recombination of photoinduced charge carriers, and poor utilization of the full solar spectrum [12, 13, 14, 15, 16]. To address these limitations, researchers have employed porous materials for photocatalytic synthesis [17], as these materials offer distinct advantages due to their tailored structures. Since their first report in 2007 [18], Conjugated microporous polymers (CMPs) have gained wide attention due to their amorphous microporous structures, remarkable stability, and ease of functional modification. These characteristics have enabled CMPs to find applications in gas storage, light collection materials, chemiluminescence sensing, and heterogeneous catalysis [19, 20]. In addition to their well‐known applications, CMPs have recently gained recognition as a powerful platform for driving a variety of photocatalytic chemical reactions. demonstrating significant potential in the field. However, despite these advancements, several challenges must still be addressed to fully harness their capabilities, requiring further investigation: (1) Improving the accessibility of catalytic active sites, (2) design with narrow‐bandgap CMPs to enhance light absorption, and (3) achieving precise control over CMPs morphology for optimized photocatalytic performance.

**FIGURE 1:**
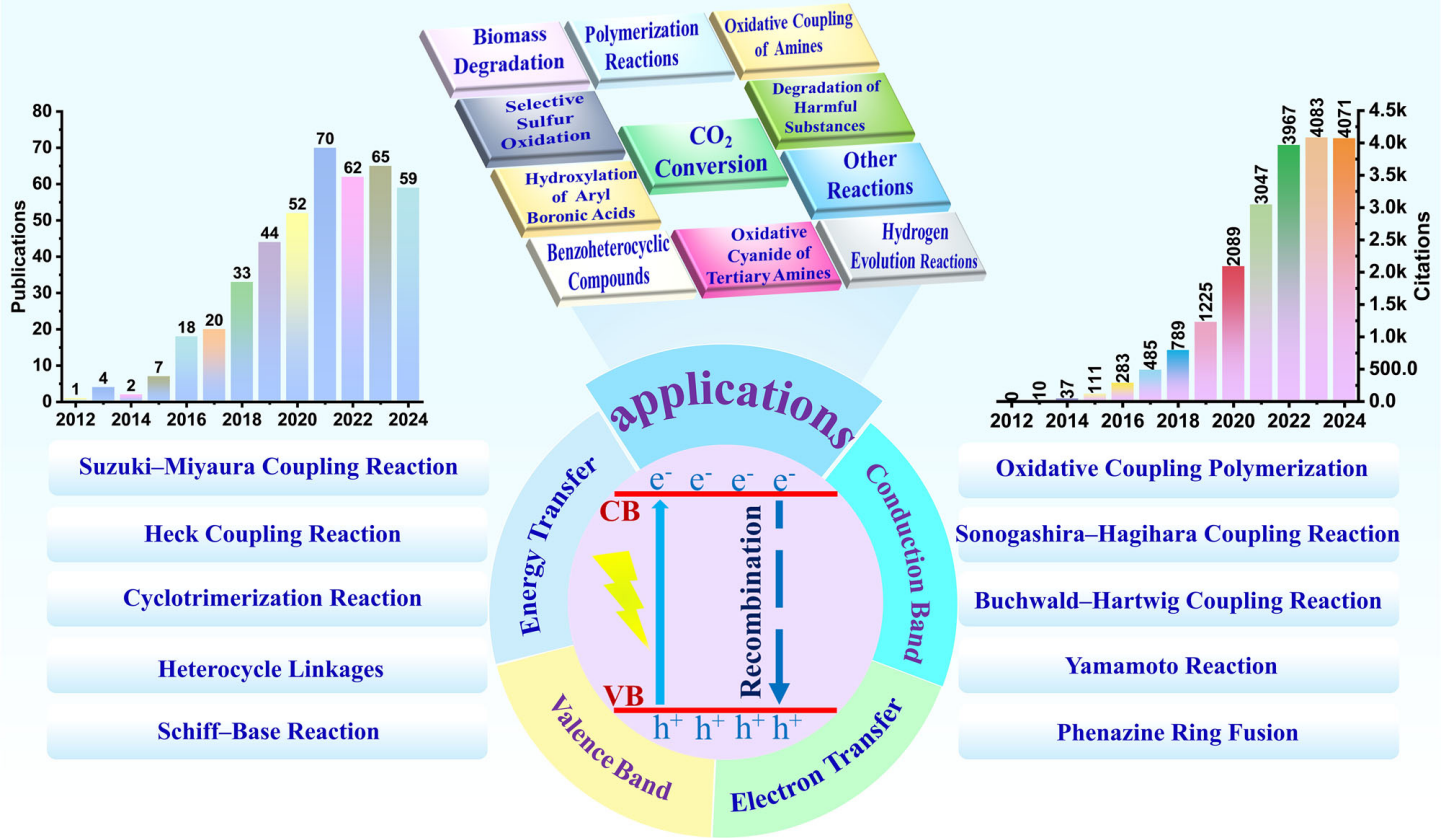
The catalytic mechanism, preparation method, application, annual publication quantity and citation rate of CMPs‐based photocatalyst (annual publications and citations on CMP photocatalysts (assessed 21st Nov 2024)).

Despite extensive research on CMPs as photocatalysts, recent advancements in the field have not been comprehensively reviewed. A time and thorough evaluation of the progress in CMP‐based photocatalysts is therefore warranted. This review summarizes recent advancements in CMPs for photocatalysis, focusing on their underlying mechanisms, synthetic strategies, and diverse applications, including hydrogen evolution reaction, CO_2_ conversion, biomass valorization, polymerization reactions, degradation of harmful substances, selective sulfur oxidation, synthesis of benzoheterocyclic compounds, cyanide oxidation of tertiary amines, aryl boronic acid hydroxylation, oxidative coupling of amines and the other reactions. A forward‐looking perspective on the key hurdles and promising avenues for the development of CMPs photocatalysts is also provided (Figure 1).

## 2 Photocatalytic Mechanism and Preparation Method of CMPs

### 2.1 Photocatalytic Mechanism of CMPs

**FIGURE 2:**
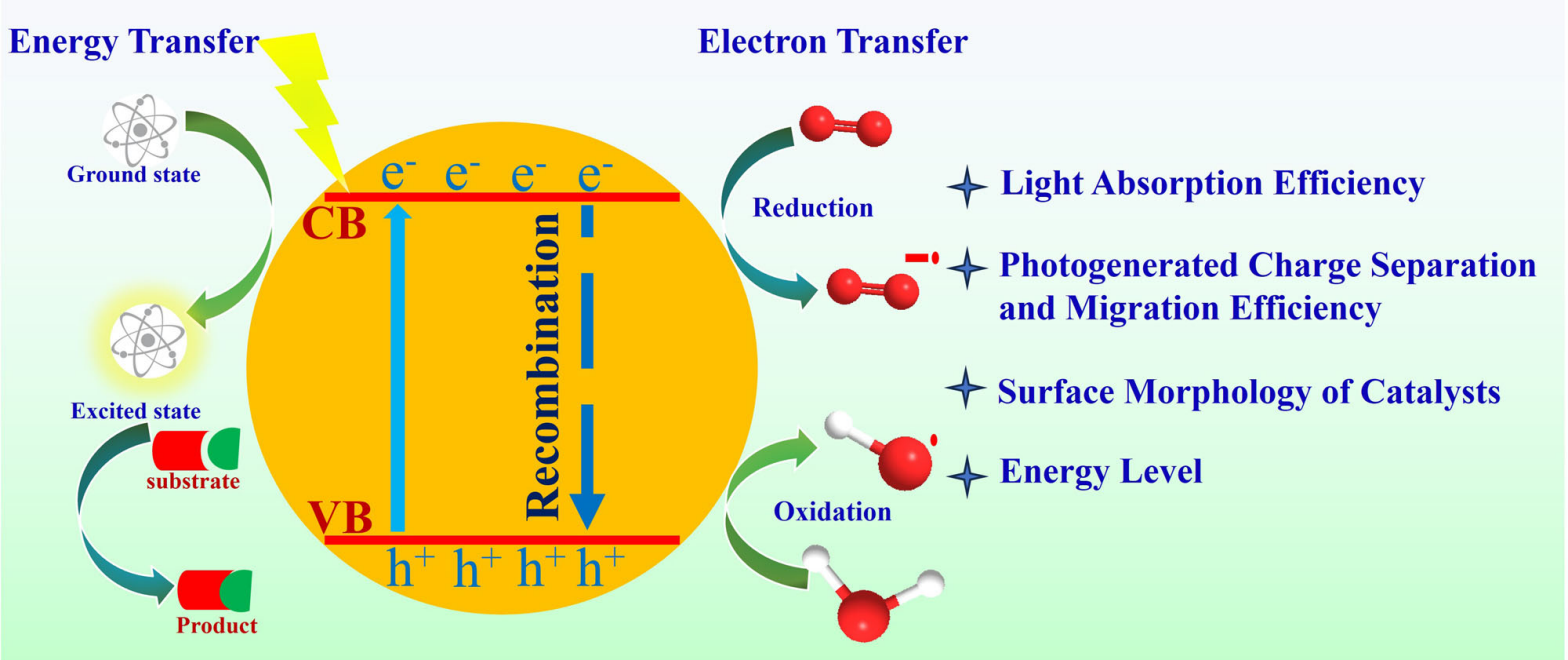
Schematic diagram of photocatalytic process and influencing factors of photocatalytic performance.

CMPs photocatalysis usually operates via three primary stages (Figure 2): (1) Light absorption: Upon absorbing photons, electrons are excited from the valence band (VB) into the conduction band (CB), producing an energized excited state. (2) Separation and migration of charge carriers: The separated charge carriers, originating within or at the surface of the catalyst, migrate to reactive sites to initiate catalytic activity. (3) Surface catalytic reaction: At the catalytic sites, the generated charge carriers engage with the adsorbed reactant species, driving chemical transformations that yield the intended products [5, 21, 22].

Enhancing the efficiency of photocatalysis remains a primary focus of research, with several strategies being employed to achieve this goal. The main approaches to improving photocatalytic efficiency include: Critical methods such as boosting photon capture [23], enhancing charge transport efficiency [24, 25], and tailoring surface morphology. Numerous methods have also been employed to eliminate these barriers, including elemental doping with nitrogen, oxygen, phosphorus, sulfur, boron, chlorine, and fluorine atoms [26], construction of heterojunctions by combining bandgap energy diversifying materials [27], utilization of donor‐acceptor (D–A) structure designs to facilitate electron transfer as well as decrease bandgaps to enable broader light harvesting [28], and utilization of cocatalysts or sacrificial molecules to hinder electron‐hole recombination. Additionally, reaction conditions, including light intensity, pH, temperature, and solvent choice, also play a crucial role in enhancing photocatalytic performance.

### 2.2 Preparation Methods of CMPs

**FIGURE 3:**
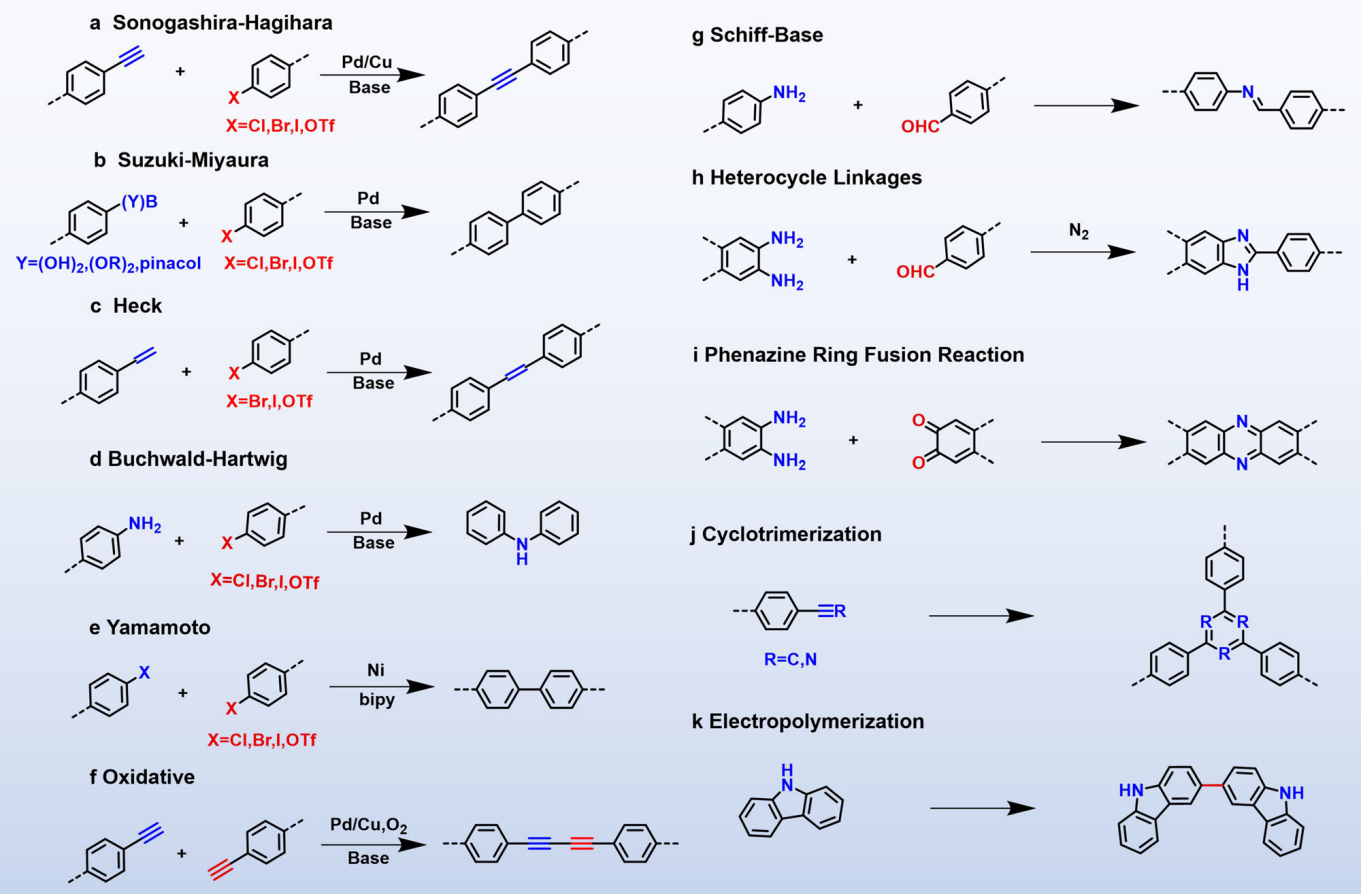
Classical synthesis route of CMPs.

The structural and functional versatility of CMPs is fundamentally governed by the selection of their constituent monomers and synthetic pathways, which directly determine the topological architecture of the polymer network and ultimately define their resulting molecular functionalities. Typically, CMPs are obtained by reacting different monomer units, or alternatively, through self‐condensation of a single monomer. Various coupling techniques (Figure 3) have been established for their construction, including Sonogashira–Hagihara [29, 30, 31, 32], Suzuki–Miyaura [33, 34, 35], Yamamoto [36], Heck [37], Buchwald–Hartwig [38], oxidative polymerization [39, 40, 41], Schiff base condensation [42, 43, 44], cyclotrimerization [45], phenazine ring fusion [46], heterocycle linkages formations [47, 48, 49], etc. These successful reaction designs have promoted the development of CMPs materials from building block design to synthesis methods, and have also developed CMPs with multiple properties, which together form the foundation of the CMPs edifice. The design, synthesis, and classification of CMPs have been extensively reviewed in recent years [19], therefore, these aspects fall beyond the scope of the current review. Instead, this review primarily focuses on the applications of CMPs photocatalysts.

## 3 CMPs‐Based Photocatalytic Applications

### 3.1 Hydrogen Evolution Reaction (HER)

Photocatalytic water splitting is one promising solution to convert sunlight into clean as well as renewable chemical fuels, which can offer a potential solution to the energy crisis as well as to environment problems [50, 51]. Hydrogen is a non‐polluting clean energy carrier that has been considered as a potentially very good energy source in the days to come [52, 53, 54]. Li et al. [55]. synthesized CMP photocatalysts in 2020 by embedding diketopyrrolopyrrole (DPP) in triphenylamine (TPA), bipyridine (bdy), as well as biphenyl (bph) building blocks to prepare DPP‐bdy‐TPA as well as DPP‐bph‐TPA architectures. DPP has superior light‐harvesting capacity due to its planar structure as well as high electron deficiency, giving rise to low bandgap as well as strong π─π stacking. The findings showed that DPP‐bdy‐TPA exhibited strong photoresponse across a broad spectrum, with apparent quantum yields (AQYs) of 9.60% at 420 nm, 7.32% at 500 nm, and 0.31% at 600 nm. Under full‐spectrum xenon lamp irradiation and visible light above 440 nm, the hydrogen evolution rates (HERs) reached 6918 µmol g^−1^ h^−1^ for DPP‐bdy‐TPA and 2780 µmol g^−1^ h^−1^ for DPP‐bph‐TPA. By comparison, it was found that the hydrophilic bipyridine unit showed better photocatalytic performance. In 2023, Meng et al. [56]. prepared pyridyl‐CMPs (*p*‐PCMP, *m*‐PCMP, *m*‐NPCMP) by polycondensation between aldehydes and aryl ketone monomers. Introduction of pyridyl nitrogen atoms enhances molecular‐level wettability in CMPs. Because of its small bandgap as well as developed hierarchical porous structure, *m*‐PCMP demonstrated outstanding photocatalytic performance in the whole ultraviolet‐visible spectroscopy (UV–Vis) region with production rates as 29.86 and 9.56 µmol h^−1^, respectively. Aside from improved wettability, pyridyl nitrogen enhances swift charge carrier mobility through enhanced transport throughout the π‐conjugated skeleton. Additionally, it facilitates tuning of photocatalytic performance through bandgap location as well as width modification.

Carbazole has good chemical and environmental stability as it is entirely aromatic [57]. Its incorporation with a bridging biphenyl unit serves to decrease the bandgap in the formed polymer, distinguishing polycarbazole from normal polyphenyl systems [58]. Ahmed et al. [59]. in 2021 synthesized two new carbazole‐thiophene‐based CMPs (e.g., Cz‐3Th and Cz‐4Th‐CMPs) by a Suzuki–Miyaura coupling reaction from characteristic coplanar carbazole precursors (e.g., Cz‐3Br and Cz‐4Br). This increased efficiency was due to the low extent of branching as well as planar molecular structure that provided increased orbital overlap in the conjugated network, thus enhancing the efficiency of photogenerated charge pair separation.

**FIGURE 4:**
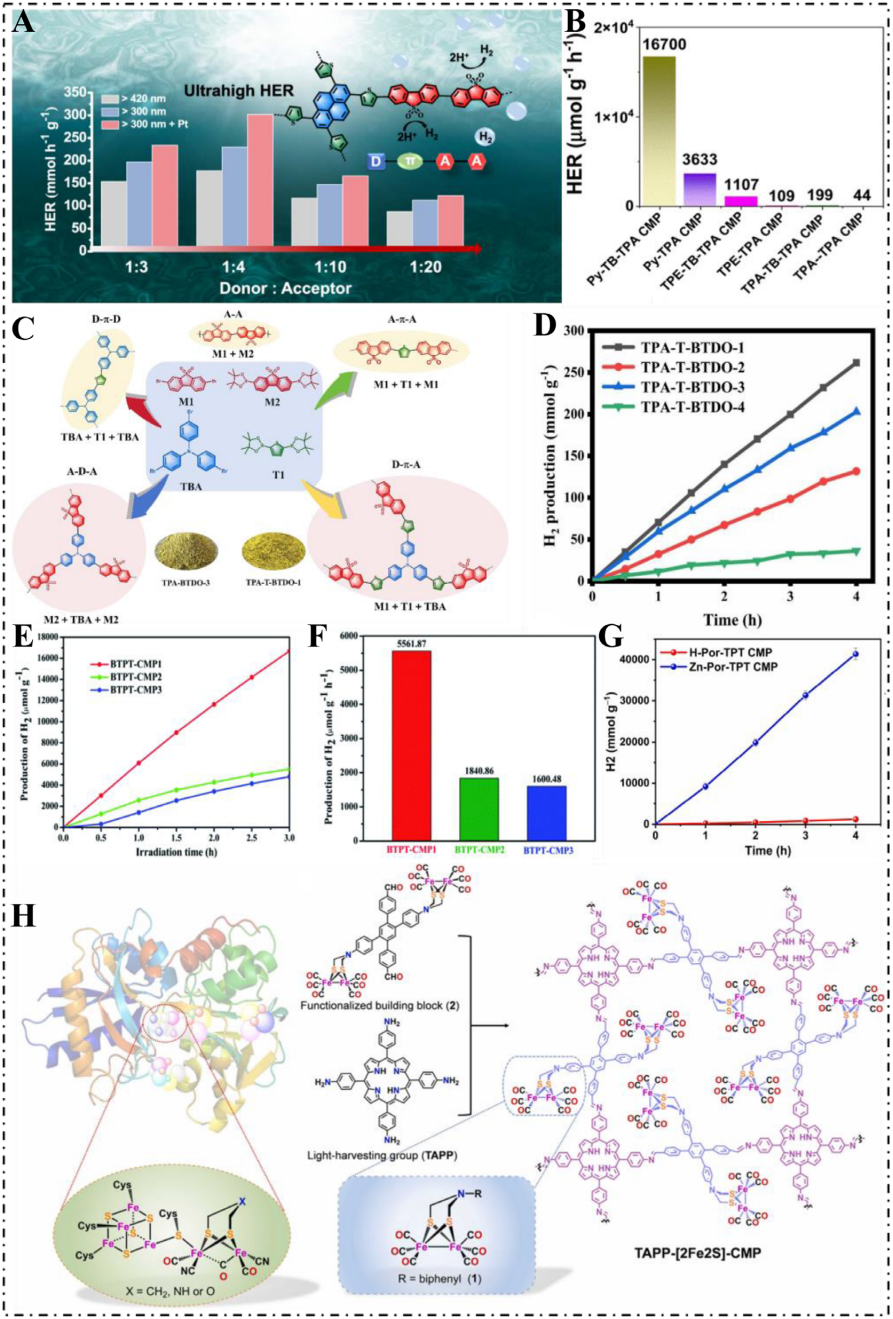
(A) Comparison of photocatalytic properties of D–π–A–A type CMPs. Reproduced with permission [61]. Copyright 2023, American Chemical Society. (B) HER profiles of TPA‐TPA, TPE‐TPA, Py‐TPA, TPA‐TB‐TPA, TPE‐TB‐TPA, and Py‐TB‐TPA CMPs. Reproduced with permission [35]. Copyright 2024, Royal Society of Chemistry. (C) Synthetic routes and possible conceptual structures of TPA‐BTDO‐3 and TPA‐T‐BDTO‐1 CMP. (D) HER rates of TPA‐T‐BTDO‐1, TPA‐T‐BTDO‐2, TPA‐T‐BTDO‐3 and TPA‐T‐BTDO‐4 under visible light (*λ* > 420 nm). Reproduced with permission [66]. Copyright 2024, Elsevier Ltd. (E) HER of the three polymers under visible light. (F) The H_2_ production rates of the three polymers. Reproduced under the terms of the Creative Commons CC‐BY license [67]. Copyright 2022, The Authors. (G) The photocatalytic long‐term stability of Zn‐Por‐TPT CMP. Reproduced with permission [68]. Copyright 2023, Elsevier Ltd. (H) The constructing the framework of TAPP‐[2Fe2S]‐CMP and the structure of its functional sites. Reproduced with permission [69]. Copyright 2023, Elsevier Ltd.

Following recent studies, dibenzothiphene‐S,S‐dioxide (BTDO) [60, 61] was discovered to be an exemplary building block for CMP‐based photocatalysts owing to the superior electron‐withdrawing nature, coplanar structure, as well as hydrophilic character of the sulfone group [62, 63]. Zhang et al. [64]. applied a statistical copolymerization method in 2021 to fine‐tune pyrene and BTDO monomers ratios in order to expand the catalytic efficiency of copolymers. An increase in the fraction of BTDO altered polymer parameters, among them the morphology, superficial area, pore size, hydrophilicity and photocatalytic efficiency. When the monomer ratio was tuned to 25:75, the material PyBS‐3 displayed strong broad‐spectrum light absorption and improved hydrophilicity, yielding a HER of 1.05 mmol h^−1^ under UV–Vis irradiation (*λ* > 300 nm) with ascorbic acid as the sacrificial donor. In 2022, Jiang et al. [65]. developed two CMP photocatalysts featuring a donor–π–acceptor (D–π–A) structure. These were synthesized using dibenzo[*g,p*]chrysene or pyrene as donor units, thiophene as the π‐bridge, and dibenzo[*b,d*]thiphene‐S,S‐dioxide as the acceptor. Among them, Py‐TP‐BTDO exhibited effective charge separation and reached an HER of 115.03 mmol h^−1^ g^−1^ under visible light (*λ* > 420 nm). Outdoor tests further confirmed its activity, with the Py‐TP‐BTDO membrane (120 cm^2^ active area) rapidly producing hydrogen bubbles under natural sunlight. In 2023, Jiang et al. [61]. synthesized a set of CMPs with D–π–A–A structures and varied BTDO contents via ternary statistical copolymerization. Among them, PyT‐BTDO‐2 exhibited the most effective performance, achieving an HER of H_2_ of 230.06 mmol h^−1^ g^−1^ (Figure 4A). This study highlights that designing CMPs with D–π–A–A structure can significantly enhance photocatalytic hydrogen production and underscores the critical role that the proportion of electron‐accepting units plays in dictating overall photocatalytic efficiency.

Due to its helical geometry—three benzene units linked to a central nitrogen—TPA is extensively used as an electron donor, recognized for its strong optical performance and excellent conductivity. As a consequence, this has widely applied in luminescent probes as well as in molecular electronics. TPA, in comparison to other aroma‐based compounds, provides wide π‐conjugation, thus supporting red‐shifted absorption as well as emission spectra. The D–π–A structure, which also enjoys superior π‐electron delocalization, can additionally enhance the efficiency in the photocatalytic process through lower exciton binding energy as well as easier exciton dissociation. In 2024, Mohamed et al. [35]. designed six CMPs originated from TPA as follows: TPA‐TPA (D–D), TPE‐TPA (A–D), Py‐TPA (A–D), TPA‐TB‐TPA (D–π–D) TPE‐TB‐TPA (D–π–A), as well as Py‐TB‐TPA (D–π–A).From these compounds, Py‐TPA as well as Py‐TB‐TPA provided HERs of 3633 mmol g^−1^ h^−1^ and 16700 mmol g^−1^ h^−1^, respectively (Figure 4B). Theoretical models exposed that through having alkyne linkers in Py‐TB‐TPA, electron–hole pair recombination can effectively decrease, charge carrier lifetime can prolong as well as transfer as well as separation efficiency can increase. A novel design strategy was presented by Zhang et al. [66]. in 2024, where the propeller‐like 3D structure of TPA acted as the donor and BTDO as the acceptor to form the macrocyclic D–A CMP catalyst TPA‐BTDO (Figure 4C). Incorporating thiophene units into the framework further enhanced planarity between the donor and acceptor components. This modification led to the development of TPA‐T‐BTDO‐1, which exhibited impressive photocatalytic HER, reaching 65.41 mmol h^−1^ g^−1^ under visible irradiation (Figure 4D). The unique twisted arrangement of TPA minimized the dihedral angle relative to BTDO, resulting in a more coplanar backbone and enhanced dissociation of photogenerated electron–hole pairs. This structural adjustment inhibits charge recombination along the polymer chain and highlights the critical role of the donor geometry in influencing photocatalytic activity.

The presence of nitrogen in the triazine unit enhances hydrophilicity, making triazine an effective electron‐accepting component for designing D–A structured CMPs aimed at photocatalytic hydrogen production. In 2022, Dong et al. [67]. developed three triazinyl D–A CMPs based on different thiophene unit crosslink lengths. BTPT‐CMP1 demonstrated superior photocatalytic capacities, in the form of blue‐shifted light consumption, enhanced photocurrent, as well as increased HER of 5561.87 mmol h^−1^ g^−1^ (Figure 4E,F). Many studies indicated that crosslink length in CMPs is very critical in relation to pushing their photocatalytic effectiveness. Mohamed et al. [70]. in 2024 outlined two new CMPs, whose designs encompassed utilization of thienyltriazine (TTh) moieties. These CMPs were tailored specifically for energy storage, as well as photocatalytic hydrogen production under visible light. Very importantly, PyT‐TTh CMP demonstrated rapid charge separation as well as low recombination losses due to which their high production capacity for hydrogen was very significant, specifically 18,533 µmol h^−1^ g^−1^ under hydrolysis conditions. In 2024, Zhang et al. [68]. developed two triazine‐porphyrin‐based CMPs—H‐Por‐TPT and Zn‐Por‐TPT. The introduction of Zinc ions significantly enhanced the photocatalytic activity, leading to a redshift in light absorption and achieving a HER of up to 11450 µmol h^−1^ g^−1^ under visible light (Figure 4G). Yan et al. [69]. reported the synthesis of another CMP, TAPP‐[2Fe2S]‐CMP, via Schiff base condensation using a [FeFe]‐hydrogenase model complex. With a HER of 2120 µmol h^−1^ g^−1^ (Figure 4H), this photocatalyst shows considerable promise for developing sustainable and high‐efficiency systems that minimize reliance on nonrenewable resources.

### 3.2 CO_2_ Conversion

The accelerated pace of industrialization and human activity has resulted in the extensive use of fossil fuels, significantly elevating greenhouse gas emissions—particularly carbon dioxide—which has triggered severe energy and environmental challenges. Taking inspiration from natural photosynthesis, researchers have designed artificial systems that harness solar energy to transform carbon dioxide (CO_2_) into valuable products such as methane (CH_4_), methanol (CH_3_OH), and formic acid (HCOOH) [71], while simultaneously driving water oxidation to release O_2_ [72, 73]. These artificial photosynthetic approaches are considered among the most effective strategies for reducing CO_2_ emissions. Among photocatalysts for CO_2_ conversion, inorganic semiconductor materials such as titanium dioxide (TiO_2_) [71, 74] and cadmium sulfide (CdS) [75] have been widely investigated for the realization of the CO_2_ photoreduction process. For this technology to reach commercial viability, substantial enhancements are still needed in the photocatalyst's light absorption capabilities, product selectivity, and overall solar energy conversion efficiency.

**FIGURE 5:**
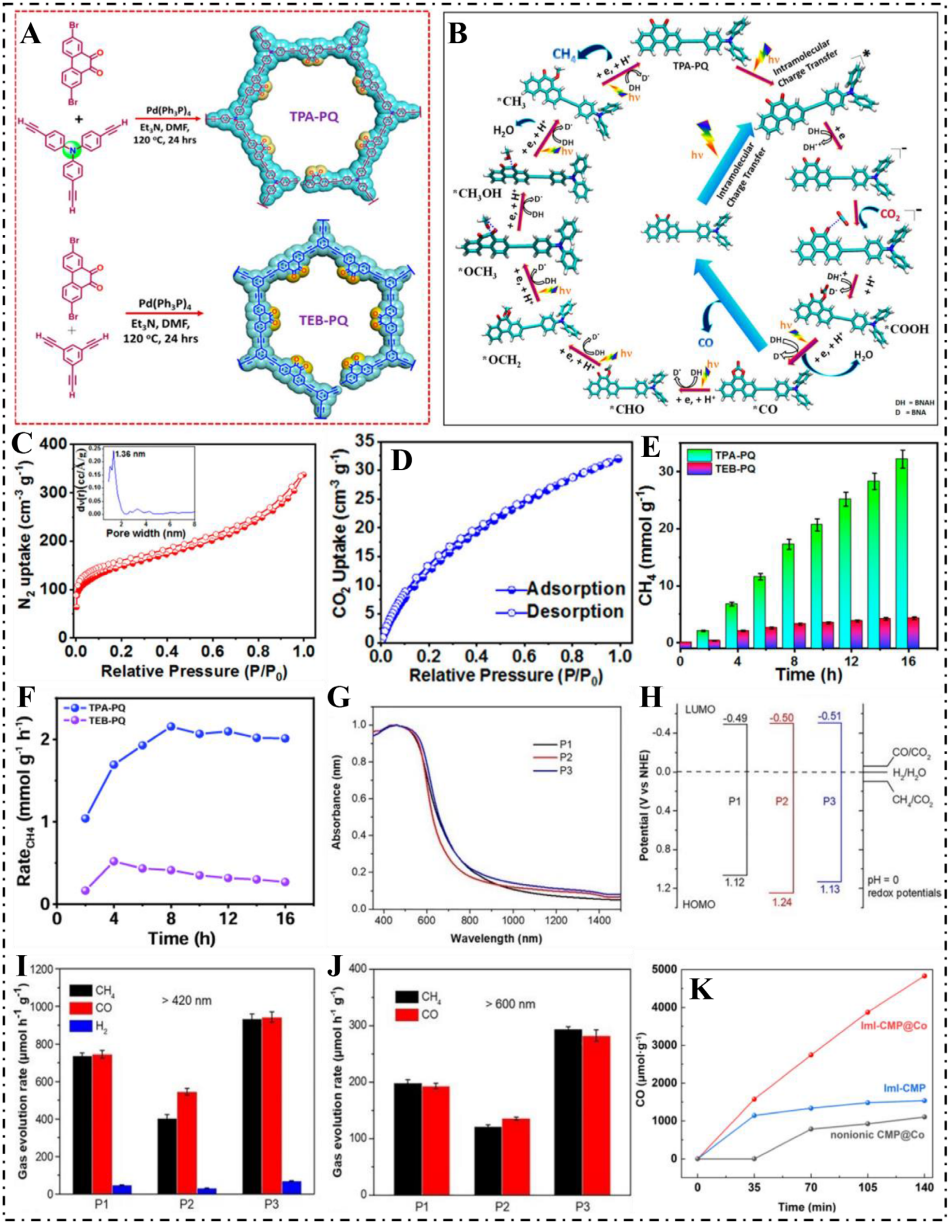
(A) Illustration of synthesis pathways of TPA‐PQ and TEB‐PQ via C─C coupling reactions. (B) Proposed catalytic mechanism depicting key intermediates involved throughout the photocatalytic cycle. (C) Adsorption isotherm of TPA‐PQ at 77 K for N_2_ (in the inset; pore size distribution). (D) CO_2_ adsorption isotherm of TPA‐PQ at 298 K. (E) Time‐dependent formation of CO_2_ reduction products in MeCN‐H_2_O (F) Comparison of CH_4_ production rates between TPA‐PQ and TEB‐PQ under similar reaction conditions. Reproduced with permission [76]. Copyright 2021, American Chemical Society. (G) Normalized UV–vis–NIR absorption spectra. (H) Band alignment of the three polymers at pH = 0. Photocatalytic activity of the CMPs for CO_2_ reduction upon (I) visible light and (J) red light irradiation. Reproduced with permission [77]. Copyright 2022, Wiley‐VCH. (K) Photocatalytic evolution of CO by ImI‐CMP@Co, ImI‐CMP and nonionic CMP@Co. Reproduced with permission [78]. Copyright 2021, Elsevier Ltd.

To maximize solar spectrum utilization, Soumitra et al. [76]. in 2021 designed a metal‐free photocatalyst that operates under visible light to reduce CO_2_ into methane. they successfully synthesized a redox‐active CMP named TPA‐PQ (Figure 5A). To elucidate its photocatalytic mechanism, density functional theory (DFT) calculations were performed (Figure 5B). The material's porosity was confirmed using Brunauer–Emmett–Teller (BET) analysis, which revealed a surface area of 492 m^2^ g^−1^ (Figure 5C). Additionally, TPA‐PQ achieved a CO_2_ uptake of 30 cm^3^ g^−1^ (Figure 5D), indicating its strong potential for use in photocatalytic CO_2_ fixation and transformation. The excellent CO_2_ adsorption capacity makes TPA‐PQ and TEB‐PQ ideal candidates as non‐homogeneous media suitable for CO_2_ photoreduction. TPA‐PQ demonstrated exemplary visible‐light‐driven CO_2_ reduction performance, producing 32.2 mmol g^−1^ of methane in 16 h (Figure 5E), with maximum turnover number (TON) as high as 40.5 in the same time period. The rate of production for methane was up to as much as 2.15 mmol g^−1^ h^−1^, with selectivity over 97% (Figure 5F). Low levels of hydrogen were also found to accumulate in the process of the reaction. This article outlines a promising avenue towards designing extremely efficient heterogeneous photocatalysts, offering practical solutions to environmental challenges as part of the general solution to the world energy crisis.

To broaden the scope of applications and light absorption capabilities of CMP materials, in 2022, Wu et al. [77]. constructed three CMPs with intramolecular D–A structures (P1, P2, P3) by combining electron‐donating pyrene and electron‐accepting fluorenone derivatives in a polymer network. The three polymers exhibited significant light absorption over a broad spectral range of 350 to 1000 nm (Figure 5G) and had a narrow energy gap of 1.61 to 1.74 eV (Figure 5H). By employing triethanolamine (TEA) and 1‐benzyl‐1,4‐dihydronicotinamide (BNAH) as sacrificial agents, the P3 CMP demonstrated the highest photocatalytic activity under visible light, achieving carbon monoxide and methane production rates of 932.9 and 943.4 µmol h^−1^ g^−1^, respectively (Figure 5I). Under red‐light irradiation, P3 continued to perform effectively, yielding CH_4_ and CO at rates of 293.7 and 282.6 µmol h^−1^ g^−1^ (Figure 5J), with nearly complete reaction selectivity. The enhanced performance of P3 over P1 and P2 is linked to its greater CO_2_ uptake capacity and more effective photoinduced charge transfer. This study provides an encouraging avenue to the development of CMPs responsive to wide spectra for sunlight‐efficient CO_2_ conversion.

**FIGURE 6:**
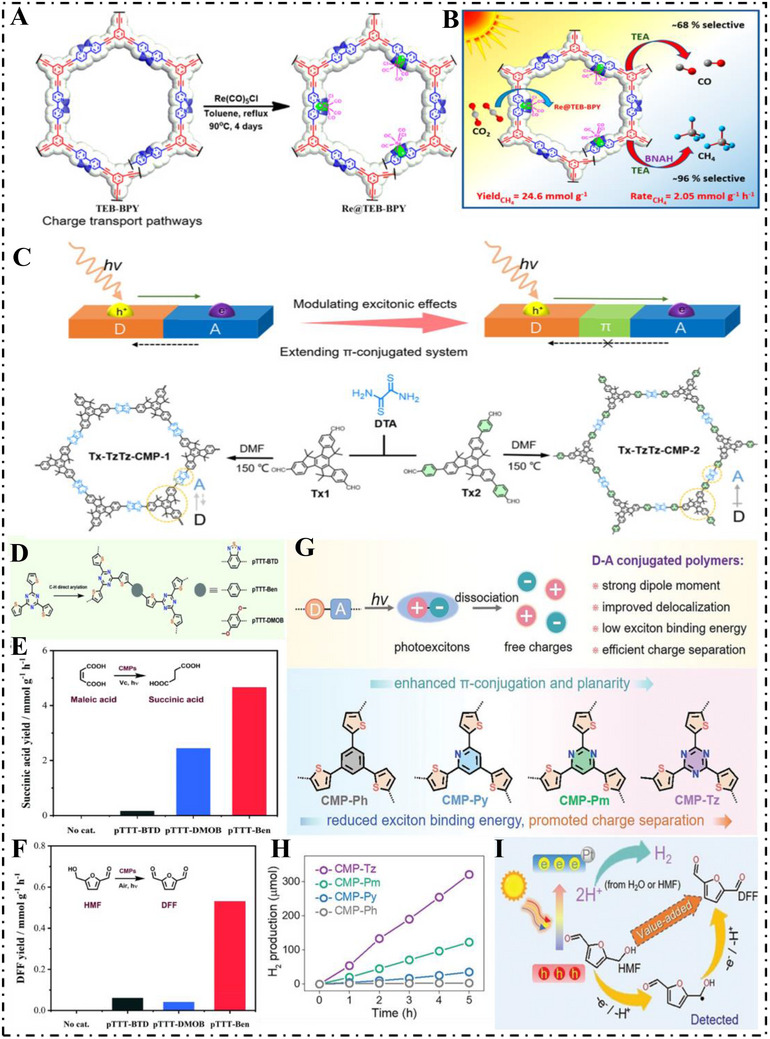
(A) Synthetic scheme for the preparation of Re@TEB‐BPY. (B) General overview of photocatalytic CO_2_ reduction based on Re(I)‐Integrated CMPs. Reproduced with permission [79]. Copyright 2023, American Chemical Society. (C) Schematic diagrams of photoexcited charge transport paths of Tx‐TzTz‐CMP‐1 and Tx‐TzTz‐CMP‐2 materials based on D–A and D–π–A systems and their synthetic routes. Reproduced with permission [80]. Copyright 2023, American Chemical Society. (D) Synthetic routes of CMPs. Photocatalytic production rates of succinic acid (E) and DFF (F) with different catalysts. Reproduced with permission [81]. Copyright 2021, Royal Society of Chemistry. (G) Schematic diagram of the design of CMPs. (H) Photocatalytic H_2_ production of CMPs in the presence of 0.1 m ascorbic acid. (I) Proposed reaction mechanism for the oxidative conversion of HMF to DFF. Reproduced with permission [82]. Copyright 2023, Wiley‐VCH.

Besides the light‐harvesting ability of the photocatalytic material, in CO_2_ photoreduction, structure design on CMP also affects efficiency. Zhao et al. [78]. in 2022 designed a new series of ion‐CMPs from imidazolium salts (ImI‐CMPs), which enhanced CO_2_ reduction under visible light dramatically by boosting intramolecular charge transfer (ICT). Complementing the structure of ImI‐CMPs with Co(II) ions enhanced performance even more to reach carbon monoxide (CO) production rates up to 2953 µmol g^−1^ h^−1^ as well as turnover frequencies up to 30.8 h^−1^ (Figure 5K). DFT calculations confirmed that easy CO_2_ activation owing to imidazole moieties in conjunction with rapid photoinduced electron transfer (PET) facilitated through π‐conjugation as well as inner electric field in polymer controlled performance greatly. This research opens powerful strategy avenues towards sophisticated organic photocatalytic designs through cationic imidazole substructure integration into conjugated systems as well as points to the cooperative role that metal ion integration can contribute towards enhancing CO_2_ photoreduction efficiency. Rahimi et al. [79]. synthesized TEB‐BPY CMPs by C─C coupling polymerization and prepared Re@TEB‐BPY metal hybrid polymers for photocatalytic CO_2_ reduction utilizing the coordination ability of bipyridine with [Re(CO)_5_Cl] (Figure 6A). Employing TEA as a sacrificial donor resulted in CO formation at 91.7 µmol g^−1^ h^−1^ with 68% selectivity. Substituting BNAH as the sacrificial agent and maintaining TEA as the base redirected the reaction toward methane, achieving 2.05 mmol g^−1^ h^−1^ with approximately 96% selectivity and a quantum efficiency of 0.22% (Figure 6B). Mechanistic insights were obtained through in situ diffuse reflectance Fourier transform infrared spectroscopy (DRIFTS) analysis and DFT modeling. From these studies, as well as controlled experiments, it is understood that TEA is extremely crucial in back electron transfer suppression when BNAH is added, consequently enhancing the overall efficiency in the photocatalytic process. This work improved the CO_2_ photoreduction yield by introducing metal ions, which provides the basis and theoretical foundation for the synthesis of metal‐hybridized CMPs.

The photocatalytic reduction of CO_2_ to CH_4_ not only needs to be considered with high conversion efficiency but also should ensure high selectivity to reduce the generation of by‐products. In 2023, Meng et al. [80]. achieved a CO_2_ photoreduction selectivity of 71.2% through rational adjustment of the donor as well as acceptor structure in the CMP structure. To establish an effective donor–π–acceptor (D–π–A) framework, the researchers strategically synthesized two redox‐active truxene based CMPs—Tx‐TzTz‐CMP‐1 and Tx‐TzTz‐CMP‐2—incorporating thiazolyl [5,4‐*d*] thiazole linkers (Figure 6C). Of the two, Tx‐TzTz‐CMP‐2—with its extended π‐conjugated—delivered a methane yield of 300.6 µmol g^−1^ h^−1^. BET analyses also confirmed their large surface area of 654 and 676 m^2^ g^−1^, respectively. Experimental measurement as well as theory calculation confirmed that conversion from D–A architecture to D–π–A structure can effectively drive ICT as well as carrier separation. Apart from Apart from that, the nitrogen atom in the thiazole group acts as electron reservoir as well as catalytic site to facilitate CO_2_ activation as well as reduced hydrogenation to produce methane. This study offers feasible guidance to rationally engineer the photo‐catalysts with high CO_2_ to CH_4_ conversion selectivity.

### 3.3 Biomass Valorization

Biomass serve as a renewable source of organic material that can be converted intonumerous valuable chemical products. Although precious metal catalysts are frequently utilized in such catalytic processes, their widespread application—combined with the need for elevated temperatures and pressures—leads to high energy demands and increased operational costs. [83, 84, 85] Consequently, the development of intelligently designed photocatalysts, utilizing sunlight as the catalytic energy source, offers a promising approach to replacing the harsh reaction conditions and costly catalytic redox processes traditionally associated with biomass valorization [86].

In 2021, Ma et al. [81]. synthesized a group of CMPs by linking 2,4,6‐(tri‐2‐thiophenyl)‐1,3,5‐triazine (TTT) with three different linker units—benzothiadiazole (BTD), benzene (Ben), and dimethoxybenzene (DMOB)—producing the materials pTTT‐BTD, pTTT‐Ben, and pTTT‐DMOB, respectively (Figure 6D). These linkers significantly influenced the morphology, optical behavior, electrical conductivity, and photocatalytic activity of the resulting CMPs. These CMPs were utilized in biomass conversion processes, effectively catalyzing the hydrogenation of maleic acid into succinic acid with a production rate of 4.66 mmol g^−1^ h^−1^ (Figure 6E), and facilitating the light‐driven oxidation of 5‐hydroxymethylfurfural (HMF) to 2,5‐diformylfuran (DFF) at a rate of 0.53 mmol g^−1^ h^−1^ (Figure 6F). These results highlight the promise of carefully engineered CMPs as sunlightresponsive photocatalysts for the sustainable transformation of biomass‐based feedstocks.

In 2023, Huang et al. [82]. designed a D–A CMP photocatalyst that was optimized structurally on the atomic level to increase charge separation in addition to overall efficiency as a photocatalyst. The compound integrated thiophene as the electron donor moiety while the acceptors were aryl *N*‐heterocycles. Through tuning in nitrogen content in acceptors, the polymer was in a position to fine‐tune the electron affinity to vary the band structure in addition to the polymer's exciton binding energy. Interestingly, through embedding s‐triazine into the polymer chain, extensive D–A interaction was formed, significantly enhancing electron delocalization in addition to reducing exciton binding energy to provide superior photocatalytic performance (Figure 6G). Therefore, the resulting CMP‐Tz exhibited significantly improved charge‐separation efficiency. To study the oxidation pathway of HMF, in situ electron paramagnetic resonance (EPR) spectroscopy was employed with 5,5‐dimethyl‐1‐pyrroline‐*N*‐oxide (DMPO) as a spin trap to capture and identify reactive radicals. CMPs form and separate photoexcited holes as well as electrons on their surface under light. Electrons are transferred to a platinum cocatalyst to convert protons to gaseous hydrogen in aqueous solution. The holes, however, oxidize HMF by abstracting one electron and one proton from the α–C–H site to yield detectable HMF radicals by EPR. When tested with ascorbic acid as a sacrificial agent, CMP‐Tz exhibited a maximum HER of 3207 µmol g^−1^ h^−1^ (Figure 6H). Furthermore, CMP‐Tz enabled dual hydrogen evolution as well as extremely selective stoichiometric HMF oxidation to DFF (Figure 6I). The report presents pioneering protocols for systematic polymer‐supported photocatalytic designs that can accomplish their dual roles in solar fuel production as well as biomass‐derived chemical valorization.

### 3.4 Polymerization Reactions

Photochemistry is gaining significance as a green approach of polymer synthesis with accurate structural control, introducing spatiotemporal control of polymer chain propagation and development of complex polymeric materials [43]. Various of these photocatalytic processes have been developed to activate new chemical transformations and allow controlled syntheses of polymers of well‐defined structures using light energy. Through photoinduced polymerization, the photoexcited states of the photocatalysts dictate electron or energy transfer processes activating polymer chains directly and allowing controlled steps of initiation and termination [87]. Techniques like PET‐RAFT, atom transfer radical polymerization (ATRP), and other photoinduced radical polymerizations rely on photocatalysts to modulate both the initiation and termination stages of polymer chain formation [88].

**FIGURE 7:**
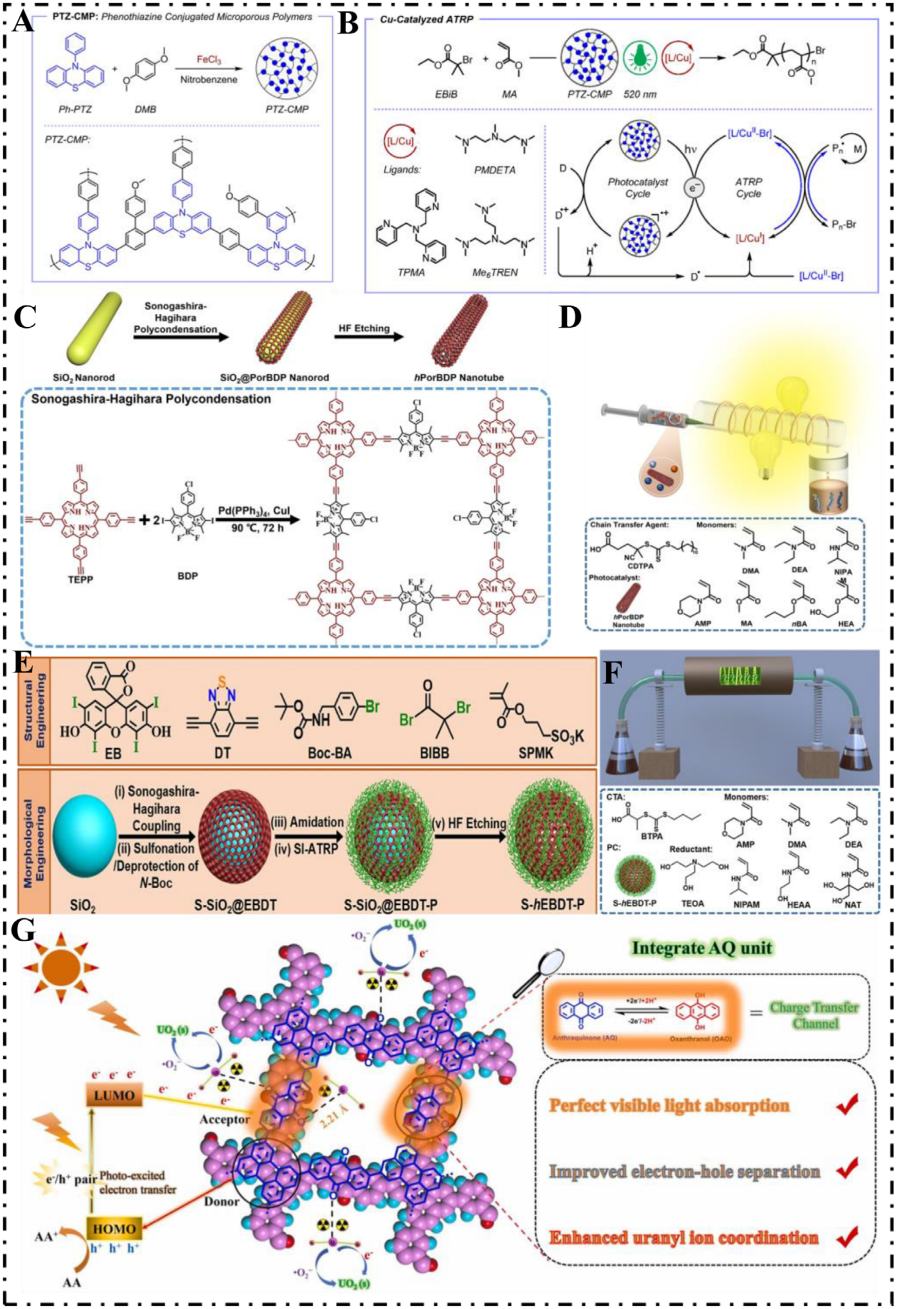
(A) Synthesis of PTZ‐CMP. (B) Mechanistic process of photo‐induced copper catalysis of ATRP using PTZ‐CMP as a heterophase photocatalyst. Reproduced with permission [89]. Copyright 2021, American Chemical Society. (C) Synthetic routes for *h*PorBDP nanotube. (D) Schematic representation of PET‐RAFT polymerization catalyzed by *h*PorBDP. Reproduced with permission [90]. Copyright 2023, Elsevier Ltd. (E) Synthetic pathway of S‐*h*EBDT‐P HCMPs. (F) Schematic representation of PET‐RAFT polymerization catalyzed by S‐*h*EBDT‐P. Reproduced with permission [91]. Copyright 2024, American Chemical Society. (G) The proposed photocatalytic U(VI) reduction mechanism of the **ECUT‐AQ**. Reproduced with permission [92]. Copyright 2022, Elsevier Ltd.

For effective use of long‐wavelength visible light sources in ATRP, it is essential to devise proper photocatalytic systems that can connect PET processes along with ATRP components and then activate controlled/living polymerization [44]. Matyjaszewski et al. [89]. prepared a dual catalyst system of phenothiazine‐based CMPs (PTZ‐CMPs) utilized as a heterogeneous photocatalyst and a copper catalyst utilized to manage ATRP under green and red light in 2021 (Figure 7A). Using dimethoxybenzene (DMOB) utilized as a crosslinking agent, the PTZ subunits were embedded within a photoresponsive network using a Friedel–Crafts reaction and prepared PTZ‐CMPs with visible‐light functionality based on the extended aromatic skeleton that encircles the PTZ cores (Figure 7B). Under green or red light irradiation, these of CMP‐ based photocatalysts activate Cu‐catalyzing ATRP and result in proper polymerization of methacrylate and acrylate monomers at high conversion rates and molecular weight control.

Light‐induced continuous flow reactors have emerged as a reliable and sustainable method for the production and processing of small and large molecules to facilitate efficient and homogeneous irradiation pathways. Despite significant advances, available homogeneous photocatalysts are still constrained by weaknesses that prevent widespread adoption of this technology [93]. To overcome these weaknesses, Cai et al. [90]. prepared hollow nanotube CMPs (*h*PorBDP NTs) of silica nanorod sacrifice templates using Sonogashira–Hagihara cross‐coupling polycondensation in 2023. Prepared nanotubes were of hollow cores and high porosity and facilitated charge separation and abundant accessible catalytic sites for photocatalytic processes (Figure 7C,D). These CMP nanotubes were efficient catalysts and were used successfully for PET‐RAFT polymerization of acrylates and acrylamide and were found to yield excellent molecular weight distribution control. In 2024, Cai et al. [91]. challenged the low dispersibility of aqueous‐phase photopolymerizations under continuous flow by preparing hairy hollow CMPs nanocomposites (S‐*h*EBDT‐P HCMPs) via a template–directed method (Figure 7E). They revealed these HCMPs exhibited remarkable water dispersibility and strong resistance toward oxygen and were observed to serve as effective heterogeneous catalysts for accelerating PET‐RAFT reactions under batch and continuous‐flow processes. Optimum reaction conditions made it possible to repetitively use the catalyst S‐hEBDT‐P and scale up poly(4‐acryloylmorpholine) (PAMPs) productions at high output and low molecular weight distributions using flow reactors (Figure 7F). This work forms the foundation of fine‐tuning PET‐RAFT polymerization using flow processes toward developing intelligent and tunable catalytic platforms of use for high‐value polymer synthesis on a scale‐up basis.

### 3.5 Degradation of Harmful Substances

With the rapid and vigorous development of modernization, a large number of harmful substances are being produced by human activities [36, 94, 95, 96]. These hazardous chemicals are real threats to the health of humankind and can cause lasting and irreparable harm to the environment. Therefore, it is essential that those treatment processes which are not only highly effective but at the same time cost‐ and energy‐saving and eco‐friendly are developed and designed intricately. To this end, photochemistry has been considered as an ideal route for the light‐driven degradation of harmful substances.

In 2022, Chen et al. [97]. designed a visible‐light‐responsive CMP photocatalyst called pTTT‐Ben, capable of extracting U(VI) from highly acidic environments. The study examined the effects of various hole scavengers on the photocatalytic reduction process and determined that ascorbic acid was the most efficient in enhancing U(VI) removal. Its strong reduction potential and strong interaction with the basic nitrogen atoms in pTTT‐Ben likely contributed to the highest U(VI) removal efficiency observed. The photocatalyst exhibited visible light absorption in the 500–600 nm range and had an optical bandgap of 2.50 eV, enabling the reduction of U(VI) under natural sunlight. Under environmental conditions averaging 27.1°C with solar intensity at 1181.9 W m^−2^, pTTT‐Ben achieved an 82% U(VI) removal rate within 8 h. Furthermore, selectivity tests involving 11 competing metal ions demonstrated that pTTT‐Ben maintained good selectivity, achieving 60% U(VI) removal under light irradiation. The exceptional stability and conjugated structure of pTTT‐Ben enabled it to sustain efficient photocatalytic reduction under highly acidic environments (pH = 1), with a removal capacity of 4710 mg g^−1^. This study demonstrated the removal of hazardous substances in a strongly acidic environment and established the fundamentals for the design of photocatalysts for CMPs adapted to harsh environments.

**FIGURE 8:**
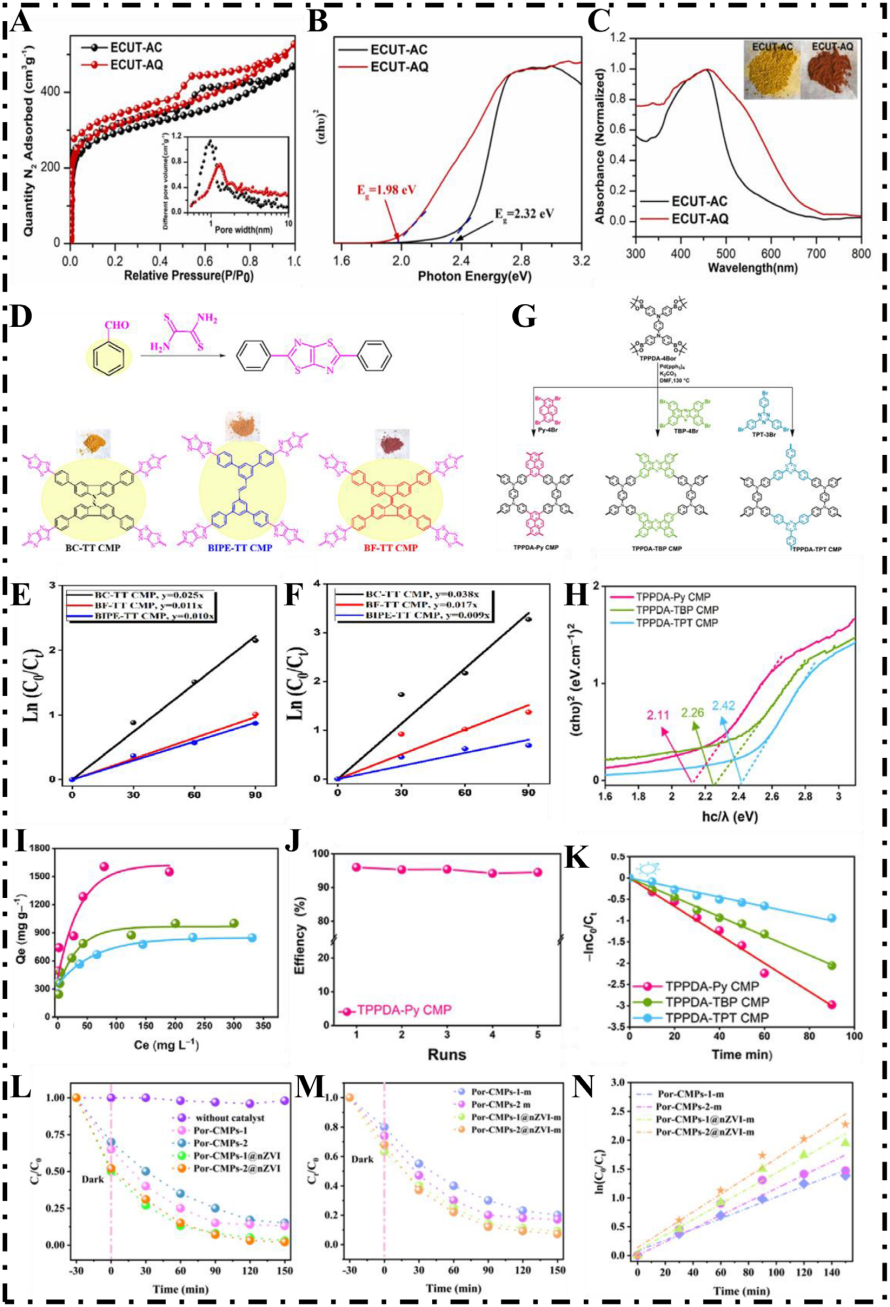
(A) Nitrogen adsorption/desorption curves at 77 K, and the corresponding pore size distribution curves of ECUT‐AC and ECUT‐AQ via NLDFT calculation (inset). (B) The bandgaps of ECUT‐AC and ECUT‐AQ calculated by Tauc‐plot. (C) UV–Vis DRS spectra of ECUT‐AC and ECUT‐AQ, inset: apparent color. Reproduced with permission [92]. Copyright 2022, Elsevier Ltd. (D) Synthetic scheme for thiazolyl‐linked CMPs. Pseudo‐first‐order kinetic curves of photocatalytic degradation of RhB (E) and MB (F) in aqueous solutions. Reproduced with permission [98]. Copyright 2023, Elsevier Ltd. (G) Synthetic routes and structures of TPPDA‐Py, TPPDA‐TBP and TPPDA‐TPT CMP. (H) Tauc‐plots calculated from UV–Vis spectra of TPPDA‐Py, TPPDA‐TBP, and TPPDA‐TPT CMPs. (I) Langmuir isothermal models of RhB adsorption onto CMPs. (J) Reuse validity of TPPDA‐Py CMP for the photodegradation of RhB aqueous solution. (K) Pseudo‐first‐order kinetics of CMPs for the photodegradation of RhB aqueous solution. Reproduced with permission [99]. Copyright 2023, Elsevier Ltd. (L) and (M) Photocatalytic effect of four different photocatalysts on MB degradation at different times. (N) pseudo‐first‐order kinetic curves of photocatalytic degradation on MB. Reproduced with permission [100]. Copyright 2024, American Chemical Society.

In 2022, Yu et al. [92]. designed a CMP photocatalyst (ECUT‐AQ) incorporating anthraquinone (AQ) as the acceptor and perylene as the donor, aiming to create a D–A system that enhance solar spectrum utilization and promotes efficient electron–hole separation. AQ with strong electron‐deficient character and redox ability is the essential functional group of ECUT‐AQ and works significantly within its photocatalytic performance (Figure 7G). Nitrogen adsorption–desorption studies at 77 K confirmed that the material had a high BET surface area of 1157.6 m^2^ g^−1^ (Figure 8A). Its bandgap, as determined by Tauc analysis, was 1.98 eV, which is lower than the 2.50 eV observed in the CMP previously described by Chen et al. [97]. Additionally, it showed broad absorption from 400 to 800 nm, with a strong peak at 537 nm (Figure 8B,C). ECUT‐AQ achieved a uranium (U(VI)) removal of 73% at photoirradiation at pH =  4 and was still featured with 65% efficiency when it was challenged under highly acidic media (pH =  1) under exposure of 60 min. Compared with this, only 15.3% of uranium was removed when it was challenged under darkness and 49.7% of the removal was contributed by the photocatalytic reduction. It is suggested that reduction of AQ led to oxoanthraquinone (OAO) formed under acidic media and then gets reoxidized back to AQ when it came into contact with cavities formed by the material. This redox cycle enables hole consumption and prevents electron–hole recombination and is assisted using a sacrificial agent. This work proposed a new D–A based CMP with exceptional uranium reduction ability when it is challenged under extreme acid media and it is a useful guide of designing effective metal‐free photocatalysts that can direct wastewater treatment of radioactive wastewater.

Organic dyes dissolved in water are notoriously difficult to eliminate and can severely pollute aquatic environments [101]. In 2023, Kuo et al. [98]. proposed a synthetic strategy for crafting thiazole‐linked CMPs incorporating bicarbazole, difluorene, and biphenylene units—named bicarbazole–thiazole‐thiazole (BC‐TT), bifluorene‐thiazole‐thiazole (BF‐TT), and biphenylene–thiazole‐thiazole (BIPE‐TT) CMPs, respectively (Figure 8D). These CMPs demonstrated high porosity, with a BET surface area of 522 m^2^ g^−1^, and excellent thermal stability, confirmed by *T*_d10_ = 460°C and a carbon yield of 67%. Among the synthesized materials, BC‐TT CMP exhibited outstanding adsorption capabilities for removing organic dyes such as Rhodamine B (RhB) and methylene blue (MB), with uptake capacities of 228.83 and 232.02 mg g^−1^, respectively, and corresponding catalytic rates constants of 2.5×10^−2^ and 3.5×10^−2^ min^−1^ (Figure 8E,F). EL‐Mahdy et al. [99]. developed an innovative set of D–A type CMPs based on tetraphenyl‐*p*‐phenylenediamine (TPPDA‐CMP) (Figure 8G). These materials demonstrated excellent properties, including high thermal stability (*T*_d10_ = 546°C), a carbon yield of 74.5%, a surface area reaching up to 1430 m^2^ g^−1^, and a narrow bandgap of 2.11 eV (Figure 8H). Notably, TPPDA‐ Py‐CMP showed an exceptional RhB adsorption capacity of 1587 mg g^−1^ at room temperature (Figure 8I) and achieved nearly complete photocatalytic degradation of RhB with 99% efficiency (Figure 8J) accompanied by a rate constant of 3.3×10^−2^ min^−1^ (Figure 8K). Overall, the results establish D–A type CMPs as strong candidates for photocatalytic removal of organic dye contaminants from wastewater.

The use of superoxide radicals is a reliable method for the photodegradation of hazardous substances. In 2024, Yu et al. [100]. developed two porphyrin‐based CMPs embedded with nanoscale zero‐valent iron (Por‐CMPs‐1−2@nZVI) using a combined Sonogashira–Hagihara coupling and liquid‐phase synthesis method. These materials exhibited both micro‐ and mesoporous structures. According to BET surface area analysis, the values were 181.94 and 92.63 m^2^ g^−1^, respectively, with the reduced surface area attributed to pore obstruction caused by the nZVI particles. Upon visible‐light exposure (≥400 nm), MB degradation efficiencies of 97% and 98% were obtained by the composites within 150 min (Figure 8L). In addition, to improve the recyclability and processability of the materials, the powder materials were converted into thin films, which resulted in a slight decrease in the photocatalytic performance (91% and 93%) (Figure 8M,N), but the recyclability of the materials was improved. This study validates the potential of superoxide and hydroxyl radicals in the photodegradation of MB and provides a new approach for doping with metal ions to modulate the photocatalytic efficiency of porous polymers.

### 3.6 Selective Sulfur Oxidation

Sulfoxides, essential intermediates in pharmaceuticals, agrochemicals, and bioactive chemical production, are typically prepared through selective sulfide oxidation with molecular oxygen under sustainable conditions [102]. Still, visible‐light‐mediated aerobic oxidation to sulfoxides presents significant challenges in efficiency and selectivity.

**FIGURE 9:**
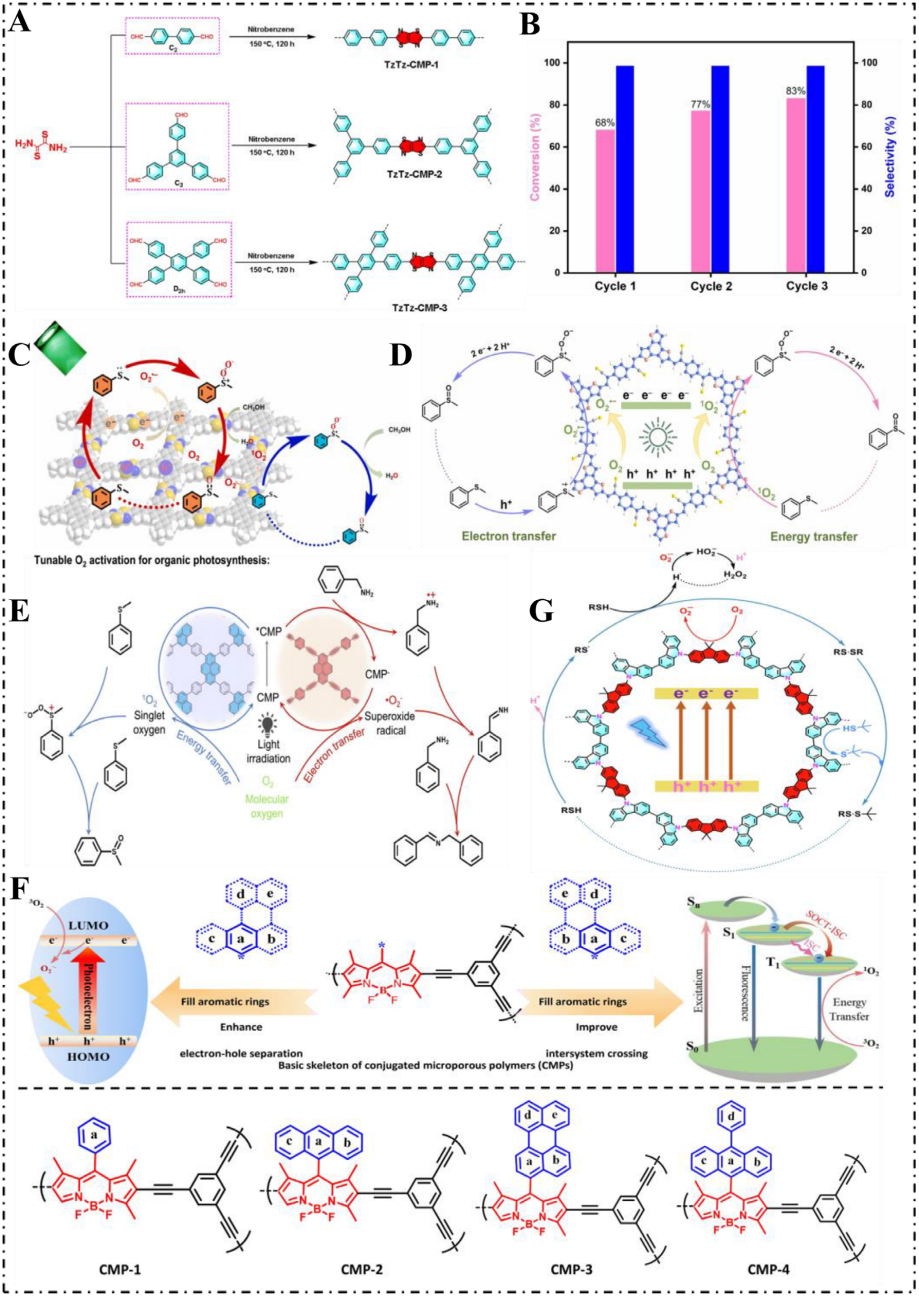
(A) The synthesis of the three TzTz‐linked CMPs. (B) Cyclic assay for the selective generation of sulfoxide by TzTz‐CMP‐3. Reproduced with permission [103]. Copyright 2021, Elsevier Ltd. (C) The plausible mechanism of Py(4)‐TzTz‐CMP photocatalysis. Reproduced with permission [105]. Copyright 2023, Elsevier Ltd. (D) Schematic of selective sulfur oxidation of BTT‐PDAN‐CMP photocatalyst with O_2_^•−^ and ^1^O_2_ under green light drive. Reproduced with permission [106]. Copyright 2024, Elsevier Ltd. (E) The mechanism of the photocatalytic aerobic oxidation reactions. Reproduced with permission [107]. Copyright 2021, Elsevier Ltd. (F) The design principles of PAHs‐modified CMPs and the structure of CMP1–4. Reproduced with permission [108]. Copyright 2021, Wiley‐VCH. (G) The possible mechanism of blue light‐induced oxidation of thiols to disulfides with oxygen on MFC‐CMP. Reproduced with permission [109]. Copyright 2022, Elsevier Ltd.

Three thiazolo[5,4‐*d*] thiazole (TzTz) linked CMP photocatalysts (TzTz‐CMP‐1,2, and 3) were prepared by Lang et al. [103] in 2021. They were designed tailor‐made with π‐conjugated aromatic rings of varying symmetry to manipulate their photocatalytic performance (Figure 9A). TzTz‐CMP‐3 was found to show the best activity toward selective sulfide oxidation under blue light exposure and the supply of atmospheric air with exceptional selectivity up to 99% (Figure 9B). The enhanced performance of TzTz‐CMP‐3 was achieved because of its highly symmetrical π‐conjugated aromatic skeleton that was accountable for higher BET surface area than their non‐symmetrically related versions. This structural feature enables more efficient conversion of oxygen to O_2_^•−^, thereby facilitating its involvement in the selective oxidation process [104]. In 2024, Lang et al. [105]. developed two CMP‐based photocatalysts incorporating TzTz linkers and investigated the impact of molecular symmetry on their performance. Among them, Py(4)‐TzTz‐CMP, featuring D2h symmetry, demonstrated superior optoelectronic characteristics compared to Py(2)‐TzTz‐CMP, which utilizes *C*_2_‐symmetric pyrene units. Under green light irradiation, Py(4)‐TzTz‐CMP achieved efficient conversion of sulfides to sulfoxides (Figure 9C). The work illustrates how structural symmetry in CMPs significantly influences photocatalytic efficiency and product selectivity, particularly for highvalue organic compounds.

Photocatalytic performance of materials is significantly influenced by their components of molecular construction, and π‐conjugated extended structures have a tendency to facilitate faster electron movement. In this work, the electron‐rich and coplanar subunit of benzotrithiophene (BTT) has attracted widespread interest. In 2024, BTT‐PDAN‐CMP and BTT‐PDA‐COF were designed and prepared by Lang et al. [106] using BTT as the core structural building block and tethered by vinylene and imine linkages, respectively (Figure 9D). Even though BTT‐PDA‐COF is a crystalline covalent organic framework (COF), the amorphous BTT‐PDAN‐CMP shows higher optoelectron performance largely due to faster electron movement facilitated by vinyl connecting linkages. For selective aerobic oxidation of sulfide applications, BTT‐PDAN‐CMP was found to be more efficient than its crystalline analogue.

When placed under green light exposure, the system permitted the development of reactive species of oxygen like O_2_^•−^ and singlet oxygen (^1^O_2_), and it effected highly selective sulfide conversion to sulfoxides. Moreover, the robust framework of BTT‐PDAN‐CMP permitted it high recyclability and general application toward green‐light‐induced sulfur oxidation reactions.

Reactive oxygen species (ROS) are key regulators in determining the mechanisms and final products of redox reactions. However, the precise quantitative structure‐activity relationship between photocatalyst design and the selective generation of ROS remains poorly understood. The generation of ROS is mainly due to differences in exciton processes, and for polymer photocatalysts, tunable molecular oxygen activation processes have rarely been reported without involving transition metal sites [110]. Therefore, it is challenging to rationally design advanced photocatalysts to modulate ROS. In 2022, Wang et al. [107] found that ROS generation can be switched by modification of metal‐free CMP, verifying for the first time the polymer‐conjugated (alkyl bridge) induced controllable molecular oxygen activation introduced in non‐metal‐CMPs (CMP‐A, CMP‐D). CMP‐A facilitates ICT to oxygens through the incorporation of an alkyne bridge and leads to O_2_^•−^ formation. However, CMP‐D lacking the alkyne linkage, facilitates the surface accumulation and diffusion of photo‐excited carriers and preferentially leads to ^1^O_2_ formation (Figure 9E). The dehydrogenation of benzamide and the oxidation of thioether were realized by two CMPs under light drive, and the catalyst loading could be reduced to 0.1% and 1%, respectively, which provided insights for the rational design of various heterogeneous organic photocatalyst CMPs.

The efficiency of photosensitization is strongly linked to a material's light absorption and capabilities and its ability to transfer energy or electrons—core processes also found in natural photosynthesis. While heavy metal atoms have traditionally been used to improve photosensitization, this strategy comes with notable drawbacks. In 2021, Guo et al. [108]. advanced the photosensitization performance of CMPs by integrating polycyclic aromatic hydrocarbons (PAHs) into their structures. By using a bottom‐up synthetic method, benzo[a]anthracene and biphenyl subunits were integrated into Bodipy‐based CMPs by Guo to prepare CMP‐2, CMP‐3 and CMP‐4. Compared to CMP‐1, both of these new CMPs had reduced fluorescence and higher effective intersystem crossing (ISC) or larger electron–hole cleavage, It was found that the yield of methylphenyl sulfoxide was 92% for the photocatalytic reaction of CMP‐2 and was markedly higher than the 32% yield of the reaction of CMP‐1 (Figure 9F). These results show that it is feasible to integrate PAHs into the backbone of CMPs and open up new channels of charge transmission and boost ISC and endow effective photocatalytic electron and energy transmission processes.

Disulfide bonds are commonly found in naturally occurring bioactive compounds, including peptides, making the selective oxidation of thiols an important chemical transformation. The formation of disulfides has gained significant attention in pharmaceutical and chemical industries [111]. Utilizing porous conjugated polymers as photocatalysts with molecular oxygen as the oxidant presents an effective approach for thiol oxidation. Nevertheless, this reaction generally depends on the combined effect of a Brønsted base and a photocatalyst to proceed efficiently. Carbazole, due to its Brønsted basicity, can assist in facilitating disulfide formation, making it a promising structural component for integrating both catalytic functions into CMPs for efficient and selective thiol‐to‐disulfide oxidation [112]. In 2022, Lang et al. [109]. utilized two precursors—9,9′‐(9,9‐dimethyl‐9*H*‐fluorene‐2,7‐diyl)bis(9*H*‐carbazole) (MFC) and 9,9′‐(9,9‐difluoro‐9*H*‐fluorene‐2,7‐diyl) bis (9*H*‐carbazole) (FFC)—to synthesize MFC‐CMP and FFC‐CMP via oxidative polymerization with ferric chloride. The CMPs demonstrated the ability to convert O_2_ into O_2_^•−^ radicals, which facilitated the blue‐light‐driven selective oxidation of thiols to symmetrical disulfides. Mechanistic studies indicated that upon blue light exposure, photoinduced electrons were transferred from the carbazole units to the fluorene moiety in MFC‐CMP, facilitating the activation of oxygen into O_2_^•−^ species. Concurrently, thiol groups adsorbed on the MFC‐CMP released protons and were converted into sulfur‐centered free radicals (RS^•^), which then combined with other thiols to form corresponding symmetric disulfide compounds, while hydrogen radicals (H^•^) were simultaneously released. Subsequently, H^•^ and O_2_^•−^ combine to form perhydroxy anion (HO_2_^–^), which is further consumed to convert the resulting H^+^ into hydrogen peroxide (H_2_O_2_) (Figure 9G). The H^+^ of tert‐butyl thiol is absorbed by carbazole of MFC‐CMP, producing anions. Asymmetric disulfides were produced through an S_N_2‐type nucleophilic substitution reaction, in which symmetric disulfides reacted with tert‐butyl thiol anions. This study emphasizes the potential of integrating synergistic photocatalytic functions into CMPs to enable diverse chemical transformations.

### 3.7 Synthesis of Benzoheterocyclic Compounds

**FIGURE 10:**
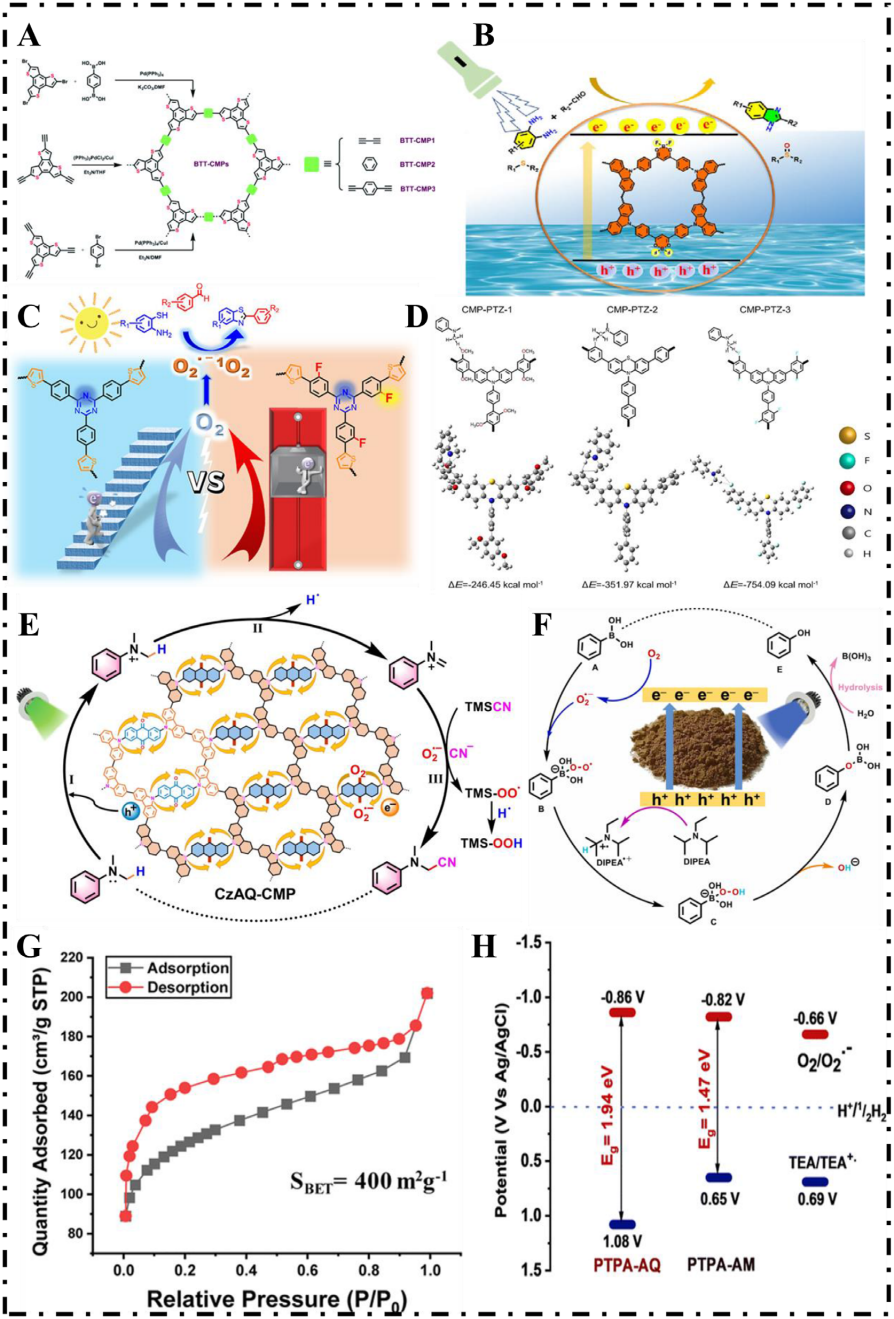
(A) Syntheses of BTT‐CMPs. Reproduced with permission [115]. Copyright 2020, Royal Society of Chemistry. (B) Catalytic mechanism diagram of Cz‐BF_2_‐CMP. Reproduced under the terms of the Creative Commons CC‐BY license [116]. Copyright 2022, The Authors. (C) Photocatalytic process of CMPs. Reproduced with permission [117]. Copyright 2023, American Chemical Society. (D) Optimized structures of hydrogen‐bonded complexes. Reproduced with permission [118]. Copyright 2020, Elsevier Ltd. (E) A preliminary mechanism for the CzAQ‐CMP catalyzed aerobic oxidative cyanidation of *N*,*N*‐dimethylaniline. Reproduced with permission [119]. Copyright 2023, Elsevier Ltd. (F) Possible mechanism of EFC‐CMP aerobic hydroxylation of phenylboronic acid to phenol. Reproduced with permission [120]. Copyright 2022, Elsevier Ltd. (G) Nitrogen adsorption‐desorption isotherms of PTPA‐AQ at 77 K. (H) HOMO–LUMO positions of the D–A CMPs. Reproduced with permission [121]. Copyright 2023, Royal Society of Chemistry.

Benzimidazole rings are ubiquitous within biologically active molecules and are vital intermediates and co‐ligands that are employed within organic synthesis [113]. Traditional routes of syntheses of benzimidazole derivatives favor harsh conditions and are therefore quite undesirable. Compared with these traditional routes of syntheses, photocatalytic synthesis is a cleaner, softer, and environmentally friendly alternative [114]. In 2020, An et al. [33]. employed the bottom‐up approach synthesizing two of their CMPs—BTP‐CMP and TBTP‐CMP—with dithiophene and thiophene components embedded, both of which were endowed with exceptional photocatalytic capacity for synthesizing benzimidazole. The electron‐rich thiophene moieties within the π conjugated TP‐CMPs contributed to beneficial band structures and boosted photochemical activity. Through the use of BTP‐CMP under illumination of visible light, more than 91% yield of benzimidazole was achieved. It was found to possess exceptional reusability (i.e., 15 cycles with yields of 96%–98%) and robustness and was a steady and highly effective heterogeneous photocatalyst. In 2021, Liu et al. [115]. prepared three conjugated porous polymers—BTT‐CMP1, BTT‐CMP2, and BTT‐CMP3—constructed from benzo[1,2‐b:3,4‐b′:5,6‐b″]trithiophene (BTT) units. They tuned the linkers between BTT moieties and achieved bandgap engineering and tailoring of the energy levels and optoelectronical features of the materials (Figure 10A). BTT‐CMP2 was found to exhibit the optimum light‐activated charge generation, transfer, and separation and produce O_2_^•−^ best. This material was proven to exhibit remarkable catalytic capacity for benzimidazole synthesis under illumination.

Boron‐based chromophores, being electron‐deficient, are viewed as effective photocatalyst candidates because of their remarkable electron‐accepting abilities, high light absorption, strong fluorescence, and elevated quantum efficiencies. In 2022, Gong et al. [116]. incorporated boron containing dyes units into the framework of CMPs to synthesize two novel materials: Cz‐BF_2_‐CMP and Cz‐BPh_2_‐CMP (Figure 10B). Through strong donor–acceptor interactions between carbazole and boron‐based dyes, these CMPs enhanced interfacial charge transfer and separation of photogenerated carriers. Thus, Cz‐BF_2_‐CMP displayed excellent photocatalytic properties, particularly in water‐based green synthesis of benzimidazoles and aerobic thioether oxidation in ethanol. In a similar context, 2,6‐dimethyl‐4*H*‐pyran‐4‐ylidene‐malononitrile (DCM), known for its excellent chemical stability and strong electron‐accepting nature, has gained attention as a fluorescent chromophore. It is widely applied in developing narrow‐bandgap photoactive CMPs due to its favorable optoelectronic properties. In 2024, Liu et al. [48]. successfully synthesized two novel sp^2^ carbon bonded DCM‐CMPs (DCM‐CMP1 and DCM‐CMP2) by Knoevenagel reaction using DCM and aromatic polyaldehydes as monomers, which have wide photoadsorption, good durability, and excellent optoelectronic properties. Notably, DCM‐CMP2 serves as a metal‐free photocatalyst that demonstrates strong catalytic efficiency, wide substrate tolerance, and outstanding recyclability in the visible‐light synthesis of benzimidazoles. This research effectively expands the utility of CMPs as heterogeneous photocatalysts, and paves the way for developing advanced solid‐state photocatalysts derived from luminescent dye‐based architectures.

Benzothiazole and its derivatives constitute a significant family of heterocycles found abundantly in nature and utilized largely in pharmaceuticals, agriculture, and industry. The photocatalytic synthesis of these molecules is of significant environmental value [122]. In 2023, Zhang et al. [117]. developed two D–A type CMPs, namely CMP‐Th‐Ph and CMP‐Th‐Ph‐F) incorporating triazine units. By integrating fluorine atoms into the CMP framework, they successfully created dual active sites involving both fluorine and nitrogen atoms and enhanced oxygen adsorption and electron and energy transfer processes (Figure 10C). These polymers selectively produced O_2_^•−^ and ^1^O_2_ Under green light illumination. Under photocatalytic conditions, 2‐aminothiophenol and benzaldehyde were oxidized and transformed to benzothiazole intermediates. This study not only introduces a novel approach for activating oxygen through organic semiconductor‐based photocatalysts but also contributes to advancing exciton manipulation strategies aimed at optimizing photocatalytic performance.

### 3.8 Oxidative Cyanide of Tertiary Amines

The oxidation of tertiary amines is a fundamental biochemical transformation that plays a crucial role in biological systems. As key precursors to nitrogen‐containing compounds, tertiary amines have garnered significant interest in organic synthesis. Traditionally, their direct cyanation has required metal‐based catalysts under elevated temperatures, and there have been limited studies using multiphase photocatalytic systems for this purpose. With their characteristic physicochemical advantages, CMPs hold significant potential for use in photocatalytic oxidative cyanation of tertiary amines [123]. In 2021, Zhang et al. [118]. introduced a series of PTZ‐CMPs as fully organic photocatalysts capable of facilitating this reaction under visible light and ambient conditions. Through altering electron‐donating and electron‐withdrawing groups on the monomers, the researchers successfully regulated charge separation and enhanced photocatalytic efficiency. Notably, CMP‐PTZ‐3, which features fluorine substituents, exhibits excellent catalytic activity and recyclability—attributed to the formation of C‐F^…^H‐X interactions between the polymer and substrate that enhance reaction efficiency. DFT calculations revealed that CMP‐PTZ‐3 had the strongest interaction energy (−754.09 kcal mol^−1^), compared to CMP‐PTZ‐2 (−351.97 kcal mol^−1^) and CMP‐PTZ‐1 (−246.45 kcal mol^−1^), suggesting stronger hydrogen bonding (Figure 10D). This work introduces a novel class of metal‐free multiphase photocatalysts and presents a refined molecular design approach for CMP‐based photocatalysis.

To investigate the synergistic interaction between electron‐deficient AQ and electron‐rich carbazole for facilitating continuous electron flow in CMPs and enhancing photocatalytic efficiency, Lang et al. [119] synthesized a CMP named CzAQ‐CMP in 2023. This material was obtained through homogeneous polymerization of a monomer (CzAQ) composed of carbazole (donors) and AQ (acceptor). Theoretical calculations validated that the AQ units within the photocatalyst promote electron transfer to molecular oxygen, resulting in the formation of O_2_^•−^. The porous architecture of CzAQ‐CMP plays a vital role in enhancing its photocatalytic performance for aerobic oxidative cyanation of *N,N*‐dimethylaniline in methanol (Figure 10E). In addition, in acetic acid medium, it catalyzes oxidative cyanation of different primary amines with excellent selectivity of up to 99%. This study introduces a novel approach and catalyst system for the application of CMPs in environmentally sustainable organic synthesis.

### 3.9 Hydroxylation of Aryl Boronic Acids

Phenols and their substitutes are common in various natural products and intermediates and are highly significant parts of polymers, pharmaceuticals, agricultural chemicals, and other bioactive entities. It is nevertheless still a challenging task to achieve direct hydroxylation of aromatic C‐H bonds. On the other hand, it is easier to achieve hydroxylation when pre‐functionalized aromatic substrates such as aryl halides or phenylboronic acids are utilized based on more accessible reactive centers. Visible photocatalysis of great interest in the selective production of phenol from aryl halides or aryl boronic acids using oxygen as an oxidant. Among the various ROSs, O_2_^•−^ is particularly well‐suited for the selective aerobic hydroxylation of aryl boronic acids to produce phenols. In 2022, Lang et al. [120]. developed two carbazole‐based CMPs (TCB‐CMP and EFC‐CMP) via C‐C coupling polymerization using 1,3,5‐tri(9*H*‐carbazole‐9‐yl)benzene (TCB) and 9,9′‐(9,9‐diethyl‐9*H*‐fluorene‐2,7‐diyl)bis(9*H*‐carbazole) (EFC) as building blocks. Under blue light irradiation and using *N*,*N*′‐diisopropylethylamine (DIPEA) as a hole scavenger, both CMPs were capable of catalyzing the aerobic hydroxylation of phenylboronic acid in ethanol (Figure 10F). EFC‐CMP outperformed TCB‐CMP in catalytic activity, which is likely attributed to its greater efficiency in reducing oxygen to O_2_^•−^, thereby enabling high‐yield hydroxylation of various phenylboronic acid substrates. In 2023, Soumitra et al. [121]. synthesized two new D–A CMPs, PTPA‐AQ, and PTPA‐AM, using Suzuki–Miyaura cross‐coupling for visible light‐driven oxidative hydroxylation of phenyl boronic acids. PTPA‐AQ exhibited a higher BET surface area of 400 m^2^ g^−1^ (Figure 10G), an optimal band gap of 1.94 eV and favorable HOMO‐LUMO energy levels, resulting in exceptional catalytic performance with phenol yield reaching 96% (Figure 10H).

### 3.10 Oxidative Coupling of Amines

**FIGURE 11:**
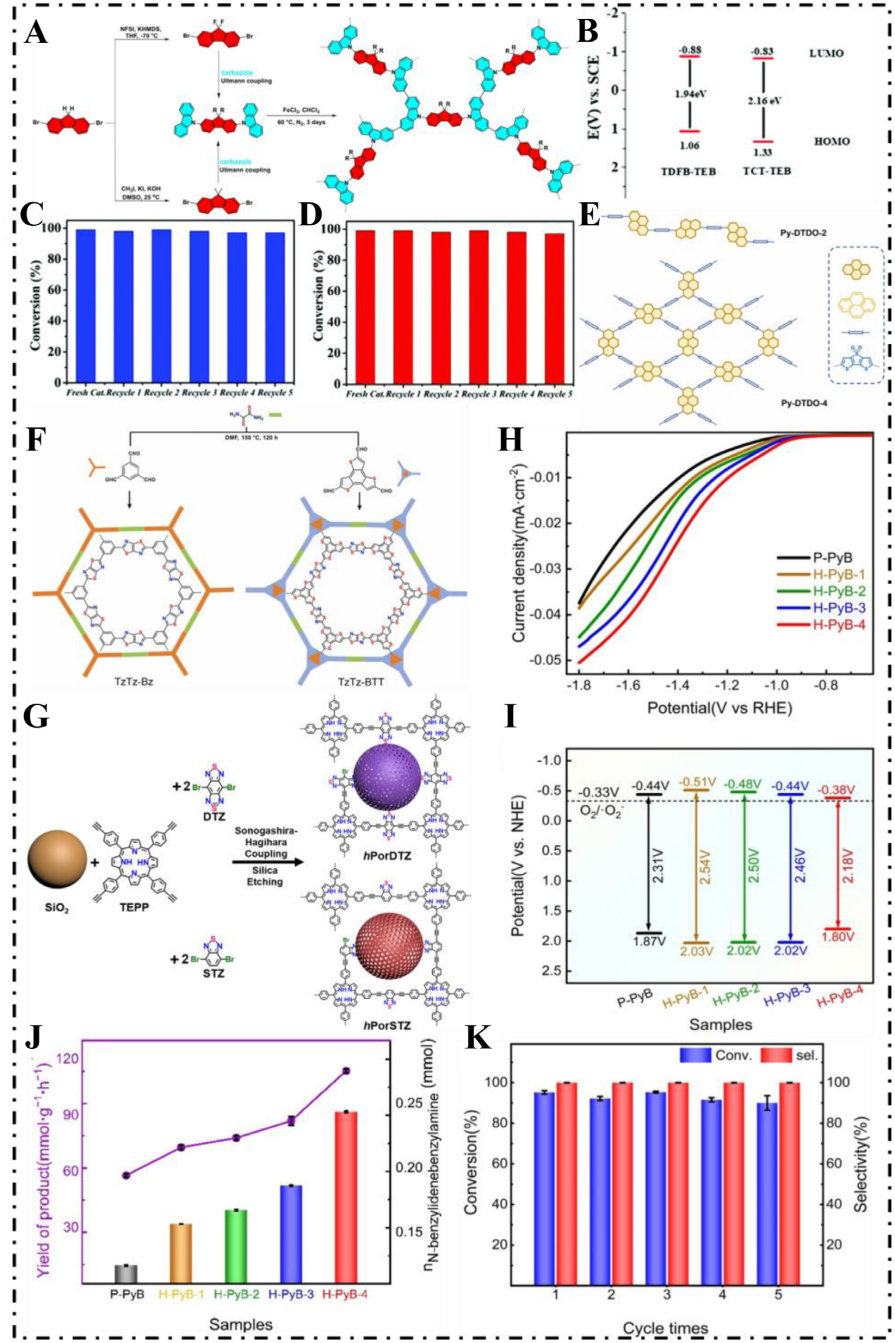
(A) Schematic of the design and preparation of two CMPs derived from fluorene. Reproduced with permission [125]. Copyright 2020, Elsevier Ltd. (B) Energy band diagrams of TCT‐TEB and TDFB‐TEB. (C, D) Assessment of the reusability of TDFB‐TEB in (C) oxidation of sulfides and (D) coupling of amines. Reproduced with permission [126]. Copyright 2021, Royal Society of Chemistry. (E) Schematic illustration for Py‐DTDO‐2 and Py‐DTDO‐4. Reproduced with permission [127]. Copyright 2022, Elsevier Ltd. (F) Synthetic routes of TzTz‐Bz and TzTz‐BTT. Reproduced with permission [128]. Copyright 2023, Elsevier Ltd. (G) Synthetic routes for *h*PorDTZ and *h*PorSTZ microspheres. Reproduced with permission [31]. Copyright 2023, Wiley‐VCH. (H) The linear sweep voltammetry curves. (I) The HOMO and LUMO energy levels derived from their optical bandgaps and cyclic voltammetry results. (J) Comparison of the yield of corresponding *N*‐benzylidene benzylamine of different photocatalysts. (K) Recycle experiments over H‐PyB‐4 CMPs. Reproduced with permission [32]. Copyright 2024, Elsevier Ltd.

Imines are very useful organic intermediates which are utilized widely in pharmaceuticals and related industries. Oxidation of amines using photocatalysts is a green and environmentally friendly approach of synthesizing imines where the reaction can occur under noncontaminating and mild conditions. In 2020, Zhou et al. [124]. prepared carbazoporphyrin based CMPs (TCPP‐CMP) by simple FeCl_3_‐catalyzed oxidative polymerization. Under natural light, TCPP‐CMP is able to promote the aerobic oxidation of benzylamine and thioanisole with high conversion and selectivity. In 2021, Lang et al. [125]. synthesized two fluorenyl‐based CMPs—MFC[9,9′‐(9,9‐dimethyl‐9*H*‐fluorene‐2,7‐diyl)bis(9*H*‐carbazole)]‐CMP, FFC[9,9′‐(9,9‐difluoro‐9*H*‐fluorene‐2,7‐diyl)bis(9*H*‐carbazole)]‐CMP—via FeCl_3_‐catalyzed oxidative polymerization (Figure 11A). Variation at the 9‐position of the fluorene methylene bridge enabled fine control over CMP photocatalytic properties. MFC‐CMP, in particular, possessed a large BET surface area and efficient electron‐hole separation, delivering outstanding amine oxidation activity under blue light.

Borane dyes are new fluorescent boron complexes of the N^B^O coordination motif and are promising building components of CMPs. Having disc‐like π‐conjugated structure with multicenter boron often results in their higher photophysical performance than ordinary dye molecules and hence are very desired building constituents of complex photocatalytic materials. In 2021, Gong's team [126] designed and successfully prepared a new boronalkyl CMP (TDFB‐TEB). In comparison to the borane‐free TCT‐TEB, TDFB‐TEB feature a narrower band gap of 1.94 eV (Figure 11B), and demonstrates excellent catalytic performance in both sulfide and primary amine oxidation reactions, achieving high conversion rates of up to 90% along with strong reusability (Figure 11C,D). In 2022, Lang's group designed six CMPs based on D–A [127] (Py‐DTDO‐2 and Py‐DTDO‐4) (Figure 11E) and D–π–A (TzTz‐CMP‐Be, TzTz‐CMP‐Py [129], TzTz‐Bz and TzTz‐BTT [128]) (Figure 11F), respectively. All of these materials exhibited excellent photocatalytic performance, and the combined findings provide meaningful guidance for the tailored development of high‐performance CMPs suited to a wide range of photocatalytic applications.

Owing to its electron‐rich character, strong charge transport ability, ease of chemical modification, and rigid π‐conjugated framework, thiophene is extensively employed as an electron donor or π‐bridge in CMP synthesis. In 2023, Zhang et al. [130]. reported the creation of two thiophene‐fused CMPs—TT‐Py‐CMP and DTT‐Py‐CMP—by coupling thieno[3,2‐*b*] thiophene (TT) and dithieno[3,2‐*b*:2′,3′‐*d*] thiophene (DTT) with 1,3,6,8‐tetrabromopyrene. Results from DFT calculations confirmed that extending the π‐system from TT to DTT strengthened visible‐light absorption and improved charge separation. Accordingly, the CMPs displayed superior catalytic activity in the selective aerobic oxidation of amines to imines. Notably, under red LED illumination, DTT‐Py‐CMP achieved a higher conversion rate of benzylamine compared to TT‐Py‐CMP.

The effectiveness of photocatalysts is often hindered by inefficient light utilization and limited substrate access to active sites. As a result, hollow CMPs have gained considerable attention due to their potential to improve catalytic performance and enable atom‐economical catalyst design through morphological tuning. [131, 132] In 2024, Cai et al. [31]. synthesized CMPs incorporating BTD units onto SiO_2_ microspheres surfaces via a Sonogashira–Hagihara coupling reaction, After removing the silica core, hollow structures—*h*PorSTZ and *h*PorDTZ—were obtained (Figure 11G). Their high surface area and modifiable surface active centers significantly boosted catalytic activity in oxidative coupling reactions of diverse primary amines. Compared to their solid counterpart (cPorDTZ), the hollow CMPs demonstrated superior reusability (over five cycles) and rapid conversion rates exceeding 90%. Xu et al. [32]. utilized dibromopyrazine and 1,3,5‐triacetylene benzene to synthesize a series of pyrazine‐based hollow spherical CMPs—H‐PyB‐1 to H‐PyB‐4—via a templating strategy, yielding structures with diameters between 630 nm and 200 nm. The confined polymerization process enhanced π‐conjugation, improved electrical conductivity (Figure 11H), reduced band gaps (Figure 11I), and enhanced optoelectronic properties. Notably, the smallest variant, H‐PyB‐4, achieved outstanding performance in photocatalytic amine oxidation, reaching a yield of approximately 113 mmol g^−1^ h^−1^ with near 100% selectivity for the resulting imine (Figures 11J and 11K). The study proposes a novel size‐limited synthesis method to design advanced organic photocatalysts, paving the way for durable, efficient, and reusable metalfree CMPs applicable to a wide range of transformations.

### 3.11 Other Reactions

Light‐driven organic transformations, for example, photocatalytic reductive dehalogenation, can constitute a very promising avenue toward green chemical syntheses. In 2023, Tang et al. [133]. developed an effective strategy for achieving highly efficient multiphase photocatalytic dehalogenation under mild conditions by modulating the exciton binding energy through variations in D–A structural frameworks. Among the materials studied, CMP‐CSU11, featuring the most optimized D–A architecture and consequently the lowest exciton binding energy, exhibited exceptional photocatalytic dehalogenation performance—achieving over 99% efficiency in approximately 50 min. It was discovered to exhibit high substrate compatibility (14 diverse substrates) and was very highly reusable and outperformed many of the available photocatalysts.

In 2023, Xu et al. [132]. constructed carbazole‐based hollow CMP spheres (H‐CTB) by sacrificial template method. Compared with the conventional CMP (P‐CTB), the hollow‐structure analogue (H‐CTB) exhibited higher physicochemical properties. H‐CTB, upon exposure to blue light, efficiently oxidized phenylethanol, producing more than 1 mmol of benzaldehyde within 4.5 h and attaining a peak rate of 9 mmol h^−1^ g^−1^, surpassing conventional CMP performance. The hollow design also facilitated oxidation reactions of other aromatic alcohols.

## 4 Summary and Outlook

Since their first demonstration in 2012, CMP‐based materials have made significant advancements in photocatalysis, showing great potential for converting photon energy into sustainable chemicals. CMPs, a subclass of COFs, constitute a new category of porous materials that integrate multiple beneficial features. These include extended π‐conjugated, outstanding optical and electronic characteristics, large surface area, intrinsic porosity, robust structural stability, tunable architectures, efficient visible‐light absorption, and excellent charge mobility—all while being metal‐free. Due to these qualities, CMPs have become a highly adaptable platform for photochemical applications, enabling diverse processes such as energy harvesting, environmental remediation, and various organic transformations.

Future developments in CMP‐based photocatalysts should focus on several key strategies to enhance their performance. One crucial area is the reduction of carrier recombination rates. This can be achieved through the construction of heterojunctions, a strategy proven effective in promoting exciton dissociation in organic semiconducting materials, as demonstrated in organic solar cells. Similarly, the integration of other semiconducting materials into CMPs can improve charge generation and photocatalytic activity. Moreover, doping CMPs with heteroatoms, such as nitrogen, oxygen, phosphorus, or sulfur allows for precise tuning of their electronic band structures, extend light absorption and promoting more efficient charge carrier separation. Surface functionalization—either through the attachment of targeted functional groups or the incorporation of metal nanoparticles—can further enhance their surface properties, adsorption capacity, and catalytic activity. The incorporation of cocatalysts, such as noble metal nanoparticles or transition metal compounds, can reduce activation energy, improving reaction rates and selectivity in photocatalytic processes.

Expanding the visible light absorption range is another key strategy for enhancing CMP photocatalytic performance. This can be achieved by incorporating chromophores or dye molecules with strong visible light absorption properties, which absorb light and transfer the energy to the polymer's conjugated backbone, improving its ability to utilize visible light. Additionally, adjusting the polymer skeleton structure by modifying monomer composition and polymerization methods can influence electron delocalization and the band structure, broadening the light absorption range.

An effective strategy to boost catalytic efficiency involve increasing the accessibility of active sites, Template‐assisted synthesis enables the fabrication of CMPs with customized morphologies and pore structures, thereby improving the exposure and accessibility of catalytic active sites. In addition, post‐synthetic modifications—such as acid, base, or redox treatments—can eliminate surface impurities and excess polymer segments, further revealing internal active sites and enhancing catalytic efficiency.

The continuous development of CMPs in photocatalysis positions them as an emerging and versatile technology for light‐driven reactions. With ongoing research focused on optimizing structural design, enhancing light absorption, and improving charge carrier dynamics, CMP‐based photocatalysts hold great promise for addressing global challenges in energy production, environmental remediation, and sustainable chemical synthesis.

## Author Contributions

Dun Zhou wrote the manuscript; Kongqing Zhang organized and collected the literature; Xiaobai Li and Hongwei Ma: project management, supervision, fund acquisition, and writing; Yongpeng Liu and Liang Yao: proofreading and supervision; this paper was jointly revised by all authors.

## Ethics Statement

This thesis does not deal with any ethical issues.

## Conflicts of Interest

The authors declare no conflicts of interest.

## Data Availability

The data that support the findings of this study are available from the corresponding author upon reasonable request.
